# A cross-institutional analysis of the effects of broadening trainee professional development on research productivity

**DOI:** 10.1371/journal.pbio.3000956

**Published:** 2021-07-15

**Authors:** Patrick D. Brandt, Susi Sturzenegger Varvayanis, Tracey Baas, Amanda F. Bolgioni, Janet Alder, Kimberly A. Petrie, Isabel Dominguez, Abigail M. Brown, C. Abigail Stayart, Harinder Singh, Audra Van Wart, Christine S. Chow, Ambika Mathur, Barbara M. Schreiber, David A. Fruman, Brent Bowden, Christopher A. Wiesen, Yvonne M. Golightly, Chris E. Holmquist, Daniel Arneman, Joshua D. Hall, Linda E. Hyman, Kathleen L. Gould, Roger Chalkley, Patrick J. Brennwald, Rebekah L. Layton

**Affiliations:** 1 University of North Carolina at Chapel Hill, Chapel Hill, North Carolina, United States of America; 2 Cornell University, Ithaca, New York, United States of America; 3 University of Rochester, Rochester, New York, United States of America; 4 Boston University, Boston, Massachusetts, United States of America; 5 Rutgers University, New Brunswick, New Jersey, United States of America; 6 Vanderbilt University, Nashville, Tennessee, United States of America; 7 University of Chicago, Chicago, Illinois, United States of America; 8 University of California-Irvine, Irvine, California, United States of America; 9 Virginia Polytechnic Institute and State University, Blacksburg, Virginia, United States of America; 10 Wayne State University, Detroit, Michigan, United States of America; University of Bristol, UNITED KINGDOM

## Abstract

PhD-trained scientists are essential contributors to the workforce in diverse employment sectors that include academia, industry, government, and nonprofit organizations. Hence, best practices for training the future biomedical workforce are of national concern. Complementing coursework and laboratory research training, many institutions now offer professional training that enables career exploration and develops a broad set of skills critical to various career paths. The National Institutes of Health (NIH) funded academic institutions to design innovative programming to enable this professional development through a mechanism known as Broadening Experiences in Scientific Training (BEST). Programming at the NIH BEST awardee institutions included career panels, skill-building workshops, job search workshops, site visits, and internships. Because doctoral training is lengthy and requires focused attention on dissertation research, an initial concern was that students participating in additional complementary training activities might exhibit an increased time to degree or diminished research productivity. Metrics were analyzed from 10 NIH BEST awardee institutions to address this concern, using time to degree and publication records as measures of efficiency and productivity. Comparing doctoral students who participated to those who did not, results revealed that across these diverse academic institutions, there were no differences in time to degree or manuscript output. Our findings support the policy that doctoral students should participate in career and professional development opportunities that are intended to prepare them for a variety of diverse and important careers in the workforce.

## Abstract 2

Will an internship during PhD training lengthen the time taken to complete the degree or hamper research productivity? Data from 10 institutions and over 1700 life science PhD trainees who participated in different amounts of professional development revealed that there were no significant differences in trainee productivity.

## Introduction

Scientific doctoral education provides technical and cognitive skill training and enables students to establish a positive sense of personal identity while building professional networks. Importantly, doctoral training provides graduates with career value in the workforce as employers increasingly recognize that employees with PhDs have advanced knowledge and skills that can enhance the organization’s productivity and reputation [1].

Three decades ago, 1 in 3 biomedical doctoral students could have expected to join the academic tenure track; however, employment trends have since shifted [2–4]. Both the National Institutes of Health (NIH) and the National Science Foundation (NSF) estimate that the current percentage of PhD scientists in tenured or tenure-track positions is less than 25% [3,5,6]. This relatively lower percentage of PhD scientists transitioning to tenure-track academic positions is ascribed to several factors. First, the number of doctoral students graduating in the biomedical sciences in the United States has steadily risen, almost quadrupling over the past 50 years [3,5,6]. Second, the growth in employment of biomedical doctoral graduates during this same time period has occurred almost entirely in industrial sectors, with comparatively little growth in employment in academic and government jobs [5–8]. Third, graduates are preferentially choosing careers in research and research-related careers beyond academia, a fact that has only recently been widely recognized by the biomedical academic community [9].

### Experiential career training

Acknowledging that a broad range of careers are pursued by PhD graduates, many doctoral programs are being redesigned or supplemented to include experiential learning and skill development to prepare students for the biomedical workforce [10,11]. Institutional efforts to supplement PhD training in preparation for varied career outcomes have been bolstered by funding opportunities from federal agencies, such as the NIH Broadening Experiences in Scientific Training (BEST) program, the NSF Research Traineeship program, and supplements to the National Institute of General Medical Sciences (NIGMS) T32 Training Grant programs [12–14]. This “value-added” training for skills such as communication, working in teams, and leadership is beneficial to those aspiring to either academic or nonacademic positions [1].

Experiential learning opportunities, including internships, allow students to consider various career paths, and these additional professional and career development activities fill gaps in research training. These opportunities equip students with skills required in the workforce, expose graduate students to different workplaces, and make them more desirable as job candidates across career types [1,2,8,15–17]. More recently, professional societies offer workshops on specialized professional development topics such as science policy and communication [18,19], entrepreneurship, and biotech careers [20] and provide other professional development programs [21]. The national call for professional and career development underscores the value of PhD-trained scientists who demonstrate a variety of skills that transcend job sectors, find satisfying careers, and contribute to the workforce, both within and beyond academia. A call to action extends beyond the biomedical arena to include the physical and social sciences, as well as the arts and humanities, and is especially relevant in light of pandemic-centered disruption to the job market and accompanying economic turmoil.

### Efficiency and productivity: Time to degree and publications

The overall length of doctoral training has long been an issue of concern. The NIH and other funding agencies, as well as policy makers, recommend exploring ways to embed career training into graduate education and postdoctoral training without increasing the time in training [5,6]. Indeed, doctoral programs struggle to shorten the time to degree, prevent attrition, and guide doctoral students to meaningful careers after training [22]. More than 85% of graduate deans surveyed in Canada, the United Kingdom, and the US have taken steps to establish supervisor guidelines to help PhD students complete their programs in a timely fashion [23]. Amid the drive to shorten doctoral training periods, a persistent and understandable faculty concern is that to add such programming during training might take focus away from the laboratory and could potentially slow research progress, which might negatively impact grant funding, publication output, and time to degree [24]. Nonetheless, data across universities show that time to degree for US students has remained relatively stable over the past 15 years [5,25].

Despite these concerns, many faculty recognize both the importance of career development to assist trainees and that their own knowledge in this area is lacking, such that supplemental programming is valuable [26]. Moreover, initiatives that have promoted professional skills to complement scientific development have shown a benefit to graduate education and have not impacted time to degree or publication output, as highlighted by individual program evaluations [27–31]. Initial data compiled from the baseline cohort of NIH BEST graduate trainees did not show a difference in average time in PhD programs over the first 3 years of data collection compared to average time before BEST implementation [8]. To further test this hypothesis, an empirical comparison was needed to examine the effects of participation in professional development on time to degree and publications across multiple institutions.

Hence, participation in career development at NIH BEST awardee institutions was examined to determine whether there were differences in time to degree as well as productivity (measured by published manuscripts) of doctoral students. BEST was an NIH grant program that funded 17 institutions across the country to develop programming that could bridge the gap between research training and the job market, a transformative effort to catalyze career development change nationally [32]. All 17 institutions were invited to participate in this study, but only 10 collected data related to program participation, publication output, and doctoral degree duration had an institutional review board (IRB) approval to share that data in this study. Our study is unique in that it compiled doctoral degree durations at these 10 universities, recorded individual participation in career and professional development activities in terms of dosage, and tracked individual engagement in real time rather than relying on surveys sent to trainees after graduation. Each of these 10 BEST institutions developed distinctive program formats and structures. Data collected from these unique programs show that there was no difference in publication output or time to degree for doctoral students who participated, even quite actively, in career and professional development activities during their academic training.

## Methods

### Participants—Institutions, programs, and trainees

#### Institutions

Participating institutions include the following: Boston University; Cornell University; Rutgers University; University of California, Irvine; University of Chicago; University of North Carolina at Chapel Hill; University of Rochester; Vanderbilt University; Virginia Tech; and Wayne State University. The institutional identifiers used herein are consistent across all figures and tables but were assigned randomly to protect institutional anonymity. These institutions include public/private, city/rural, multiple/single-campus locations, and medical school/nonmedical school settings. Institutions’ BEST programs supported populations ranging from 280 to 1,000+ doctoral trainees and 80 to 500+ postdoctoral trainees. Note that while some institutions include postdoctoral trainee participation in their BEST programs, all productivity data from postdoctoral trainees were excluded from this study because they do not have a time to degree, making it more difficult to make comparisons. Details characterizing institutional profiles, NIH BEST programs, and graduate departments/programs included in the study are provided for each institution in Tables A, B, and C in S1 Text.

Across institutions, the departments, programs, and disciplines included in this study ranged from a single biomedical PhD program to programs serving all biological and biomedical programs on a given campus; some of the institutions also include engineering, public health, or psychology disciplines (**Table A in S1 Text)**. Common programs included Molecular Biology, Genetics, Biochemistry, Biomedical Sciences, Neuroscience, to name a few—as visualized with a weighted word cloud based on participating departmental and program names (**Fig A in S1 Text)**.

#### Ethics

All 10 institutions’ studies were deemed exempt by the relevant IRB (BU IRB#: H-33268; Rutgers IRB#: E15-050; Rochester IRB#: RSRB00055304; UNC IRB# 14–0544; Vanderbilt IRB# 190288; UCI IRB#: 2014–1502; VT IRB#: 13–711; WSU IRB: #094013B3E; the remaining institutional studies were approved via IRB Exemption Protocol ID#: 1412005184 through NIH OMB #0925–0718). An individual student’s participation in the BEST program was completely voluntary, and informed consent was attained by affirming participation in program activities. Students were given the opportunity to opt out. All identifying data have been removed from raw data sets as per IRB requirements, and these data sets are available via an Open Science Framework repository (https://osf.io/qy3pa/, permanent DOI: 10.17605/OSF.IO/QY3PA; see also [33]). In the Open Science Framework repository, institutional data are in individual files. For example, the data for institution A are found in the file titled “ZA TTD data deidentified.xls,” and the data for institution B are found in the file titled “ZB TTD data deidentified.xls,” etc. Columns for each institutional data set include the following: coded trainee ID; trainee participation data and corresponding dosage bin; time to degree and/or defense; and number of publications (total, first author, and/or pub metric composite score).

#### Program activities

Each BEST institution developed its own program to achieve its program-specific goals. Program activities ranged from single events to multipart workshop series or coursework, as well as experiential learning activities, such as site visits, internships, and individual training sessions. One-off workshops were the most common activity each year for all of the programs [8]. Institutions also deployed a wide range of activities differently, allowing trainees to participate through specific phases, by sector, by career interests, ad hoc, or some combination thereof. Most institutions included experiential learning opportunities with partners outside the university. Many programs offered opportunities at their university by partnering with various professional schools, core facilities, or support offices within their institution. Another focus was on incorporating mentorship and connecting trainees to alumni and professionals in broad areas of biomedical research. From these internal and external institutional connections, a majority of the BEST institutions allowed the possibility of internships, but it was not a requirement. The BEST institutions shared strategies, activities, and contacts among the BEST network of institutions during annual NIH BEST conferences, allowing programmatic offerings to evolve over time. A more complete description of the BEST institutions’ programming can be found in Supporting information file 1 (**Tables A and B in S1 Text**).

#### Procedures

Throughout the duration of BEST funding, institutions collected data about biomedical PhD trainee time to defense and level of participation in internships and BEST activities (e.g., career panels, skill-building workshops, job-searching workshops, site visits, and internships). Data were submitted annually to NIH over a 5-year period using common forms, standardized data collection procedures, and compatible reporting methods to allow for cross-institutional comparison. Meetings to discuss evaluation of program design were held with all BEST consortium members, including a data summit to finalize common definitions and standardize BEST data collection methods (detailed collection methods including baseline data survey design and results included) [8]. Cross-institutional definitions for methods of instruction/delivery and agreements on common criteria for data were instrumental in developing the data collection methods.

The most straightforward comparison between participants and nonparticipants in BEST career and professional development programming was measurement of binary outcome differences. Hence, this was the most reliable effect size measure to use and was employed for mega-analytic comparisons. For binary comparisons using a *t* test, the no participation group (control) was compared with any participation (e.g., medium plus high participation groups), giving a sense of effect size.

We were also interested in identifying potential dose–response effects based on level of participation. As each institution offered different events with variable length and scope, each was asked to define low participation and high participation levels independently (**Table D in S1 Text**). Most institutions split their low- and high-dosage populations based on the observed median dosage level. These definitions were established so that the 3 groups could be compared, giving a sense of any dose–response effect. This additional level of analysis yielded a more nuanced ability to evaluate participation effects and query for potential negative effects on productivity when there were high levels of participation. Nonetheless, to retain the clarity of the control versus participant populations, the primary cross-institutional analysis of interest was based upon bivariate comparisons.

**Fig 1:**
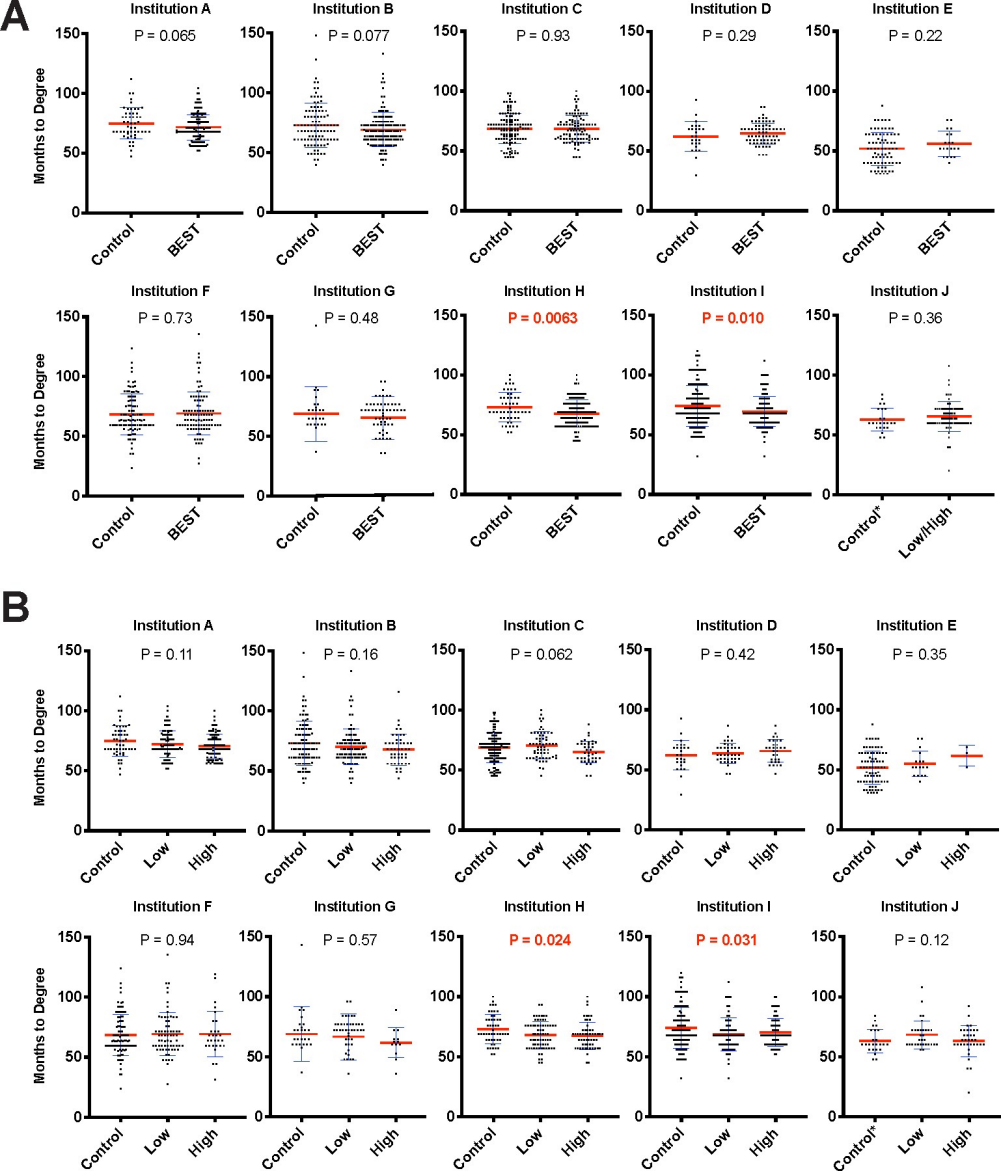
Professional development participation is not associated with an increase in time to degree. **(A)** Months to degree vs. binary professional development participation. Blue error bars represent standard deviation of the mean. Mean is denoted by a red line. Independent samples *t* tests (see Table F in S1 Text for statistical test results) were used to compare control (nonparticipants) vs. participant time to degree (significant values of *p* < 0.05 noted in red). Control* for institution J indicates that the control individuals were approximated based on available participation data (see Material and methods). **(B)** Months to degree vs. dosage of professional development participation. Blue error bars represent standard deviation of the mean. Mean is denoted by a red line. ANOVA was used to compare the impact of control, low-, and high-dose participation on time to degree (significant values of *p* < 0.05 noted in red). Control* for institution J indicates that the control individuals were approximated based on participation data (see Material and methods). The remaining participants were divided into low- and high-participation groups. All data sets are available at https://osf.io/qy3pa/ (permanent DOI: 10.17605/OSF.IO/QY3PA; see also [33]). ANOVA, analysis of variance; BEST, Broadening Experiences in Scientific Training.

For all binary analysis, with one exception, control groups were defined as nonparticipants; the exception was one program that did not have a true control group and hence divided participation in BEST events into an approximation of a control group (0 to 1 point) and a medium/high dose, rather than the null, low, and high dose used by the remaining institutions (see **Fig 1**). For consistency, the comparison groups for analysis of variance (ANOVA) are referred to as control, low, and high (control* is used to denote the approximated control group). Post hoc analysis shows no difference when this institution’s data were excluded; hence, we chose to include the data to be comprehensive.

Institutions also collected and reported publication outcomes. These data were independently gathered by each institution. Publication data were collected either by self-reported survey, manual PubMed queries, or using the PubMed API using a Python script developed for this purpose and freely provided by Hall and Arneman [34]. For those institutions that used the Python script, results were manually spot checked for potential errors (**Table M in S1 Text**), including overcounts for common names, legal name changes, nickname use, or advisor switching. In addition, extreme publication counts identified by the automated script (e.g., 0 publications or >5 publications) were manually rechecked by hand.

#### Analyses

Two approaches were used: (1) *t* tests and ANOVAs were used to compare individual institutional samples; and (2) an overall mega-analysis to compare effects across all institutions. Both methods were used to assess bivariate and dose–response effects on months to degree, total publications, and first-author publications. Binary participant/nonparticipant comparisons were evaluated using independent sample *t* tests and dose–response tests using a one-way ANOVA with a 3-level professional development dose variable (control, low, and high). Institutional sample comparisons were analyzed using Prism GraphPad (v9.0.1) software, which was also used to generate plots throughout the manuscript. Mega-analyses were performed to evaluate professional development effects overall and to generate forest plots (e.g., [35]) using SAS (v9.4; see full mega-analysis results in SI2). In accordance with mega-analysis best practices of calculating mean dosage differences, Tables H, J, and L in S1 Text display *t* tests relying on pooled error variance (allowing effects to vary between samples). The *p*-values listed in Figs 1, 3, and 5 (statistical results found in Tables F, G, I, K, P–S in S1 Text) reflect independent sample *t* tests and ANOVAs and hence differ slightly from Tables H, J, and L in S1 Text. Nonetheless, conclusions converge using both approaches.

The use of meta-analyses and mega-analyses [36,37] allows for extrapolation of an effect size and significance across different populations, multiple studies, or, in our case, different institutions and interventions. In other words, meta- and mega-analyses are used when comparing effects, especially when the variables of interest are measured differently across sites as was the case in our study (e.g., hours, events, and points). In a meta-analysis, each sample is first standardized and then the standardized effects are compared using a random effects model. Mega-analyses, on the other hand, allow variance between each sample (mimicking random effects models), but allow more granular comparison of the data, including contrasts between subgroups analogous to multigroup ANOVA. To allow for more complete intergroup contrasts, our mega-analysis incorporated both bivariate comparisons and dose–response effects. In some cases, not enough institutions were able to provide data to allow for mega-analysis (i.e., only a subset of institutions supporting internships). Mega-analyses were conducted only when a sufficient sample size was available based on a large enough set of institutions providing data (e.g., 9 to 10 studies per analysis [38]).

Primary predictors included the amount of professional development participation (binary or control/low/high dosage). Primary outcome variables of interest included productivity as measured by time to degree and publications (total and first author). Finally, all outcome measures were tested against internship participation, the highest dose of professional development implemented across sites for the subset of institutions able to provide these data.

Power calculations verified whether our sample sizes were sufficient to detect relevant effect sizes [39,40] across each mega-analysis using SAS (v9.4). For the time to degree mega-analysis, the post hoc power was calculated using a minimum effect size estimate of 3 months; for total publications and first-author publications, a minimum effect size estimate of 1 publication was used. Post hoc power analyses determined that >80% power was achieved for each mega-analysis for these effect size estimates, indicating that a sufficient number of subjects and studies were included. Exact power calculations are reported alongside the relevant mega-analyses in the results section.

## Results

### Time to degree versus professional development participation

As NIH BEST programs were implemented at each institution, some in the biomedical training community questioned whether participation in professional development programming would increase time to degree. Here, we tested this hypothesis using binary measurements (participants versus nonparticipants), as well as using a dose–response effect to determine whether higher levels of participation affect time to degree. *t* Tests were conducted for bivariate analyses and ANOVAs for multiple groups and are shown in each institution’s plot in each tile of Figs 1, 3, and 5.

Two institutions showed a statistically significant shorter time to degree for participants using either the binary or dose/level of analysis; the remaining institutions showed no significant difference in time to degree for participants in the binary condition or when accounting for level of participation (**Fig 1A and 1B)**. Some institutions collected defense dates in addition to graduation data and could therefore calculate time to defense as well. Using the measure of months to defense resulted in 2 institutions showing that greater participation was associated with a statistically significant decrease in time to defense (**Fig B in S1 Text**).

Overall, the data failed to support the hypothesis that participation in career and professional development at any level tested leads to a statistically significant increase in time in graduate training.

### Mega-analysis of time to degree versus professional development participation

**Fig 2:**
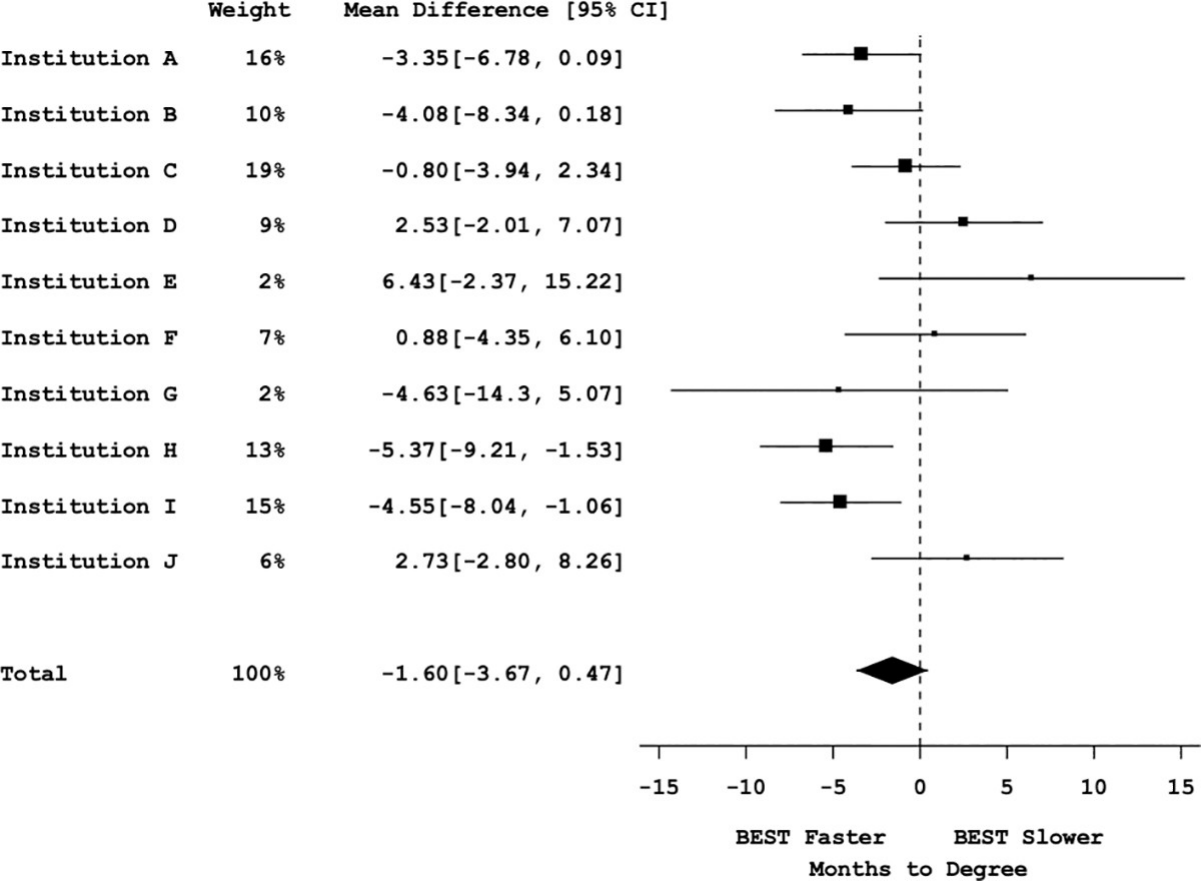
Graduate student efficiency measured by time to degree vs. bivariate participation. Mega-analysis forest plot displaying mean effect sizes (squares) and confidence intervals (brackets) for effect sizes of time to degree vs. bivariate professional development participation (control vs. participants). Large squares denote greater impact on the effect size based on sample size and effect size in each institutional sample. The vertical dotted line represents a null effect. The size and shape of the diamond at the bottom of the forest plot represent the effect size. Because the diamond overlaps the vertical line (null effect), this indicates that the effect of professional development participation on time to degree is not significant. See Table H in S1 Text for statistical results. All data sets are available at https://osf.io/qy3pa/ (permanent DOI: 10.17605/OSF.IO/QY3PA; see also [33]). BEST, Broadening Experiences in Scientific Training.

A mega-analysis was conducted to determine a weighted effect size and significance across all the institutions for the time to degree data set (**Fig 2**). This cross-site mega-analysis (including 1,742 trainees’ participation data) showed no difference in time to degree between participants and nonparticipants (point estimate of −1.60 [95% CI = −3.67, 0.47], omnibus F(2,9) = 2.66, *p* = 0.12, bivariate contrast t(9) = −1.75, *p* = 0.11 (**see Table H in S1 Text**)). Post hoc power calculations [41,42] suggest that with the sample sizes and number of participating institutions’ data included in this mega-analysis, we had 83% power (alpha = 0.05) to detect a 3-month difference in time to degree. Given that our study met the acceptable rate of 80% power [36,37], we can confidently say that we had the ability to detect an effect size of this magnitude or greater.

Furthermore, there were no cases in which the dose–response effects were significantly longer for those with the highest participation (omnibus *F* tests were not significant); in fact, in the single case of significant difference, the directionality indicated a favorable association such that participants took less time to graduate than nonparticipants. ANOVAs show comparisons between no-dose, low-dose, and high-dose event participation (**Fig 1B**).

In sum, the analysis reveals that participating in career and professional development was not associated with an increased time to degree. This finding supports the notion that participation, even in high doses, is not associated with a delay.

### Total and first-author publications versus professional development participation

**Fig 3:**
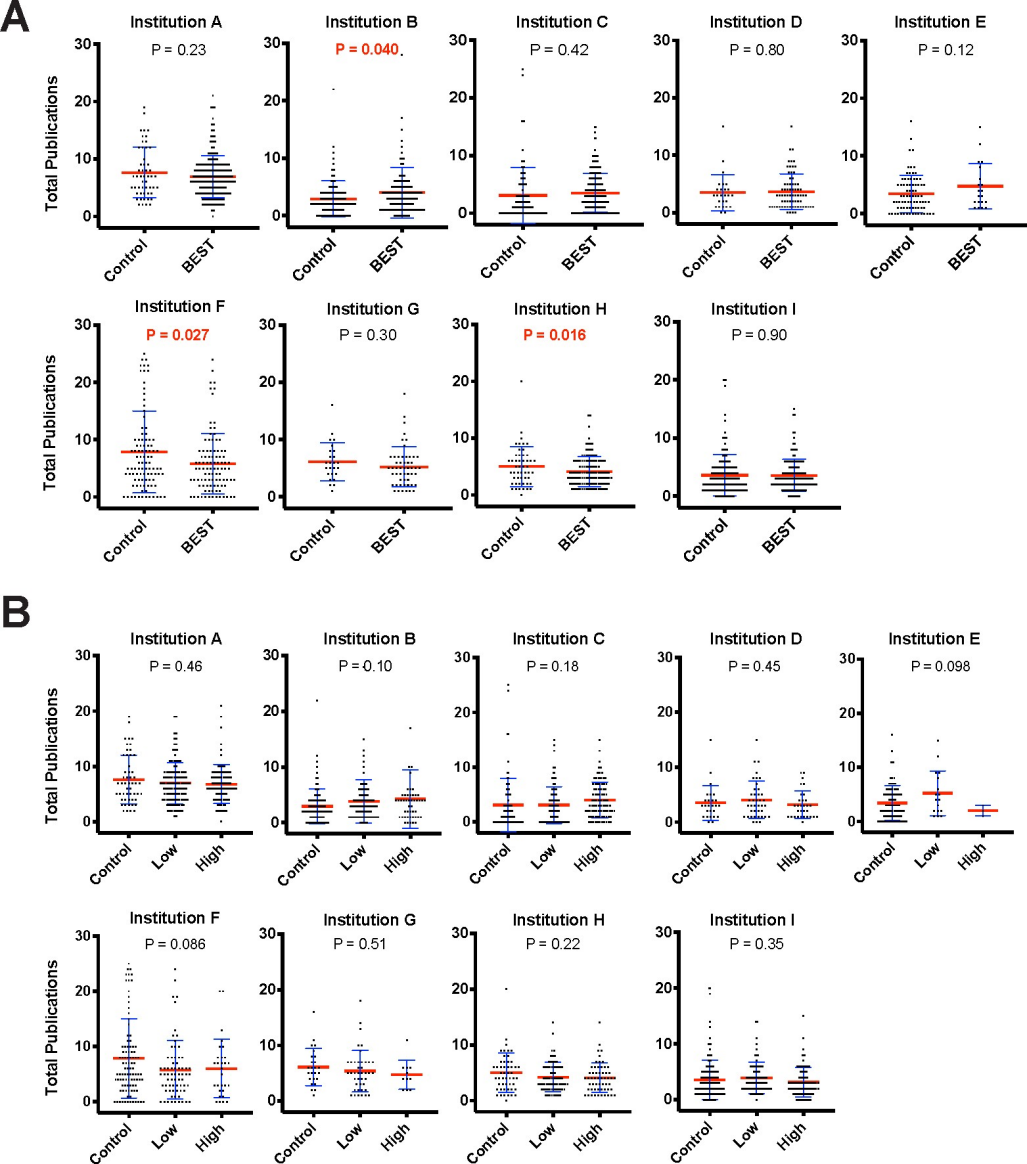
Professional development participation is not associated with a decrease in total publication. **(A)** Total publications vs. binary professional development participation. Blue error bars represent standard deviation of the mean. Mean is denoted by a red line. Independent samples *t* tests (see Table I in S1 Text for statistical test results) were used to compare of control vs. participant total publications (significant values of *p* < 0.05 noted in red). **(B)** Total publications vs. dosage of professional development participation. Blue error bars represent standard deviation of the mean. Mean is denoted by a red line. ANOVA was used to compare the impact of control-, low-, and high-dose participation on total publications (significant values of *p* < 0.05 noted in red). All data sets are available at https://osf.io/qy3pa/ (permanent DOI: 10.17605/OSF.IO/QY3PA; see also [33]). ANOVA, analysis of variance; BEST, Broadening Experiences in Scientific Training.

Next, we evaluated the impact of career and professional development participation on productivity, measured by number of publications. We first evaluated total publications during the graduate training period. For participants versus nonparticipants, 1 institution showed significantly more publications for participants, and 2 showed significantly fewer publications for participants. The remaining 6 institutions showed no significant difference between participants and nonparticipants with regard to total number of publications, and when accounting for different levels of participation, no institution showed any significant difference in the number of total publications between groups (**Fig 3A and 3B**). Furthermore, the mega-analysis contrasts and omnibus test were also not significant (see next section).

Professional scientists, faculty researchers, and doctoral training programs often place special significance on first-author publications because the bulk of trainees’ efforts in the lab are usually directed at projects resulting in first-author publications. These efforts also typically form the underpinning for the students’ theses. Due to the unique importance of first-author publications, we further examined whether there is a specific impact of participation in career and professional development on first-author publications.

**Fig 4:**
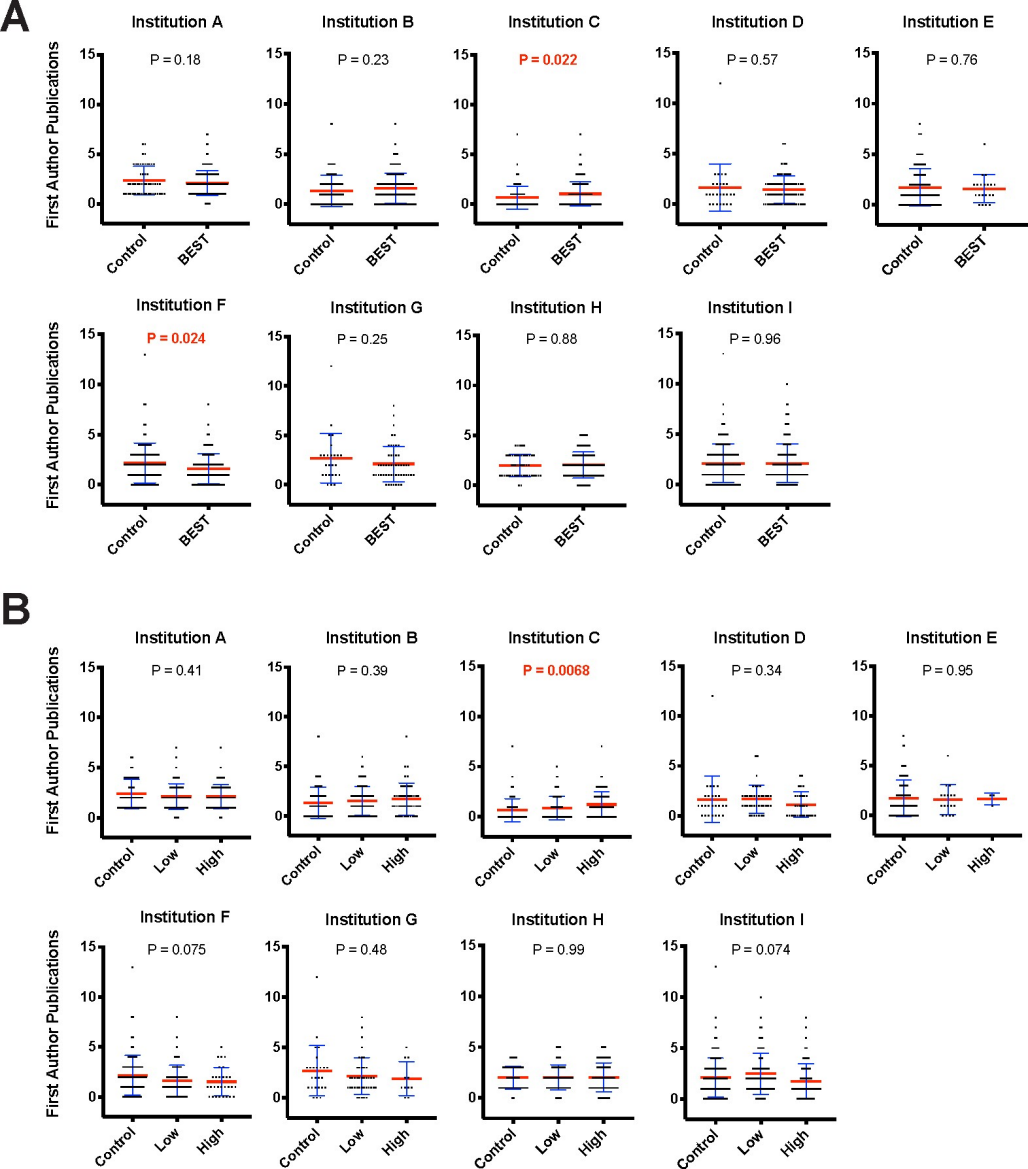
Professional development participation is not associated with a decrease in first-author publication. **(A)** First-author (including co-first author) publications vs. binary professional development participation. Blue error bars represent standard deviation of the mean. Mean is denoted by a red line. Independent samples *t* tests (see Table K in S1 Text for statistical test results) were used to compare of control vs. participant first-author publications (significant values of *p* < 0.05 noted in red). **(B)** First-author (including co-first author) publications vs. dosage of professional development participation. Blue error bars represent standard deviation of the mean. Mean is denoted by a red line. ANOVA was used to compare the impact of control-, low-, and high-dose participation on first-author publications (significant values of *p* < 0.05 noted in red). All data sets are available at https://osf.io/qy3pa/ (permanent DOI: 10.17605/OSF.IO/QY3PA; see also [33]). ANOVA, analysis of variance; BEST, Broadening Experiences in Scientific Training.

Similar to the overall number of publications, there was no conclusive effect of BEST participation on increases or decreases in, specifically, first-author publications (**Fig 4**). In the binary condition for first-author publications, one institution’s BEST participants produced significantly more first-author publications, and one institution’s BEST participants produced significantly fewer. When level of participation was considered, one institution’s “high dose” BEST participants produced significantly more first-author publications. In both the binary and dose–response analyses, the remaining institutions showed no significant difference between participants and nonparticipants in first-author publications. Accordingly, there was no overall trend of BEST participation reducing first-author publications, and the hypothesis that participation in professional development activities reduces publication rate was not supported by our data. Furthermore, the mega-analysis contrasts and omnibus test were also not significant (see next section).

### Mega-analyses of total and first-author publications versus professional development participation

**Fig 5:**
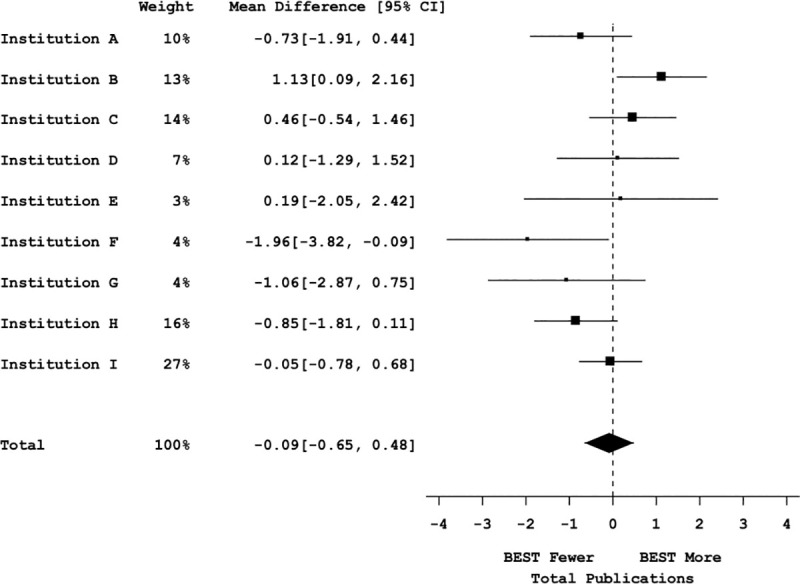
Graduate student productivity measured by total publications vs. bivariate participation. Mega-analysis forest plot displaying mean effect sizes (squares) and confidence intervals (brackets) for effect sizes of total publications vs. bivariate professional development participation (control vs. participants). Large squares denote greater impact on the summary effect based on sample size and effect size in each institutional sample. The vertical dotted line represents a null effect. The size and shape of the diamond at the bottom of the forest plot represent the effect size. Because the diamond overlaps the vertical line (null effect), this indicates that the effect of professional development participation on total publication is not significant. See Table J in S1 Text for statistical results. All data sets are available at https://osf.io/qy3pa/ (permanent DOI: 10.17605/OSF.IO/QY3PA; see also [33]). BEST, Broadening Experiences in Scientific Training.

**Fig 6:**
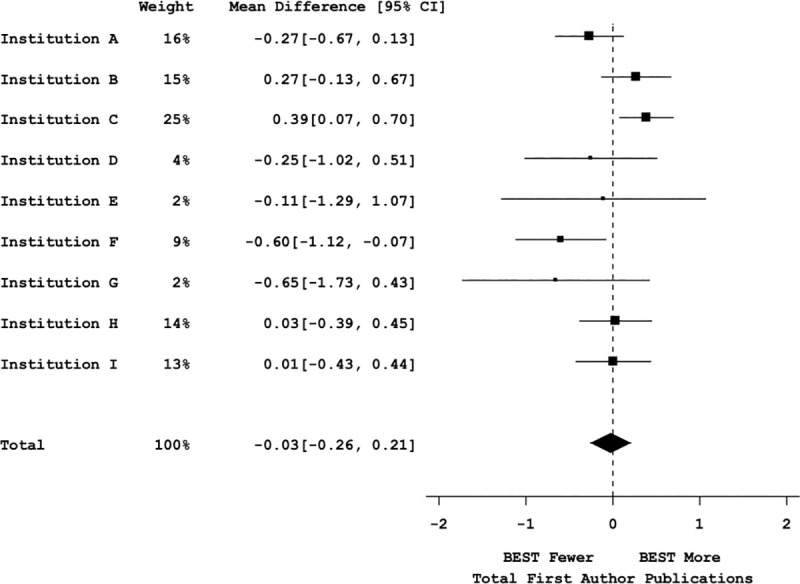
Graduate student productivity measured by first-author publications vs. bivariate participation. Mega-analysis forest plot displaying mean effect sizes (squares) and confidence intervals (brackets) for effect sizes of first-author publications vs. bivariate professional development participation (control vs. participants). Large squares denote greater impact on the summary effect based on sample size and effect size in each institutional sample. The vertical dotted line represents a null effect. The size and shape of the diamond at the bottom of the forest plot represent the effect size. Because the diamond overlaps the vertical line (null effect), this indicates that the effect of professional development participation on time to first-author publications is not significant. See Table L in S1 Text for statistical results. All data sets are available at https://osf.io/qy3pa/ (permanent DOI: 10.17605/OSF.IO/QY3PA; see also [33]). BEST, Broadening Experiences in Scientific Training.

Mega-analyses were conducted to determine the weighted effect size and significance across all the institutions for total and first-author publications (**Figs 5** and **6**, respectively). The cross-site mega-analyses (including 1,698 trainees’ publication data) showed no significant difference in total publications between participants and nonparticipants (with a point estimate of −0.09 [95% CI = −0.65, 0.48]) (see **Fig 5**; omnibus F(2,8) = 0.24, *p* = 0.79, bivariate t(8) = −0.34, *p* = 0.74 (see **Table J in S1 Text**)). Similarly, a mega-analysis of first-author publications from the same institutions showed no significant difference in first-author publications between participants, and nonparticipants (with a point estimate of −0.03 [95% CI = −0.26, 0.21]) (**Fig 6**; omnibus F(2,8) = −0.25, *p* = 0.96, bivariate t(8) = −0.25, *p* = 0.81 (see **Table L in S1 Text**)). In conclusion, across a large multi-institutional sample, there was a lack of evidence for reduced trainee productivity as measured by publication number.

Post hoc power calculations [41,42] suggest that with the sample sizes and number of participating institutions’ data included in this mega-analysis, we had more than 94% power to detect a difference of one publication (total or first author).

### Weighted publication metric (PubMetric): An alternative comprehensive publication measure

Both first-author publications and total publications capture different aspects of productivity. By choosing to report one or the other, some information is lost. Instead of limiting the accuracy of reporting by removing one or the other, we created a novel publication metric that could capture trainees’ efforts on both types of contributions in a single metric. One concern that we anticipated was how to weigh these different contributions. For instance, designation as the first author on research papers generally denotes greater effort compared to other types of contributions (e.g., middle-author research paper contributions or review papers). To address this issue, University of North Carolina at Chapel Hill (UNC) developed a weighted publication metric (**Text A in S1 Text**) that incorporates the 4 primary types of peer-reviewed publications into a single number. Impact factor was not included as a variable in the publication metric because impact factor as a measure of paper quality or journal prestige can be inherently biased by field. Additionally, citation-based impact factors are not representative of productivity for young scientists because not enough time has elapsed from the time of publication for recent graduates. The UNC-weighted publication metric was designed as a broader and more objective measure of the amount and quality of author contributions by trainees as reflected by authorship order.

To create the weighted publication metric, active training faculty at UNC were asked to rank the relative value of (A) first-author peer-reviewed research articles; (B) first-author peer-reviewed review articles; (C) middle-author peer-reviewed research articles; and (D) middle-author peer-reviewed review articles (*n =* 150 responses from 350 total contacted; see **Text A in S1 Text** for details). First-author and co-first-author publications were considered synonymous. When averaging all faculty rankings and normalizing middle-author reviews to a weighting of 1, we generated the following equation for the weighted publication metric (PubMetric).


$$\begin{array}{c} \underline{Weighted\;Publication\;Metric\;(PubMetric)}=2.07\times (number\;of\;first‐author\;research\;papers)+\\ 1.54\times (number\;of\;first‐author\;reviews)+1.37\times (number\;of\;middle‐author\;research\;papers)+\\ 1.0\times (number\;of\;middle‐author\;reviews). \end{array}$$

Four BEST institutions were able to provide weighted PubMetric data from PubMed scripts. Using this metric, similar patterns emerged as for total publications and first-author publications, namely we found no difference in publication output between participants and controls (**Fig C in S1 Text**).

### Internships, efficiency, and productivity: Time to degree, total publications, and first-author publications

Internships are a form of career training that have unique characteristics and formats but require a relatively large time commitment that one could predict would impact time to degree or productivity. Institutions that supported internship opportunities provided outcome data for trainees who participated in their internship programs, which had differing lengths and designs, and all had some variant of a competitive selection process (**Table O in S1 Text).**

**Fig 7:**
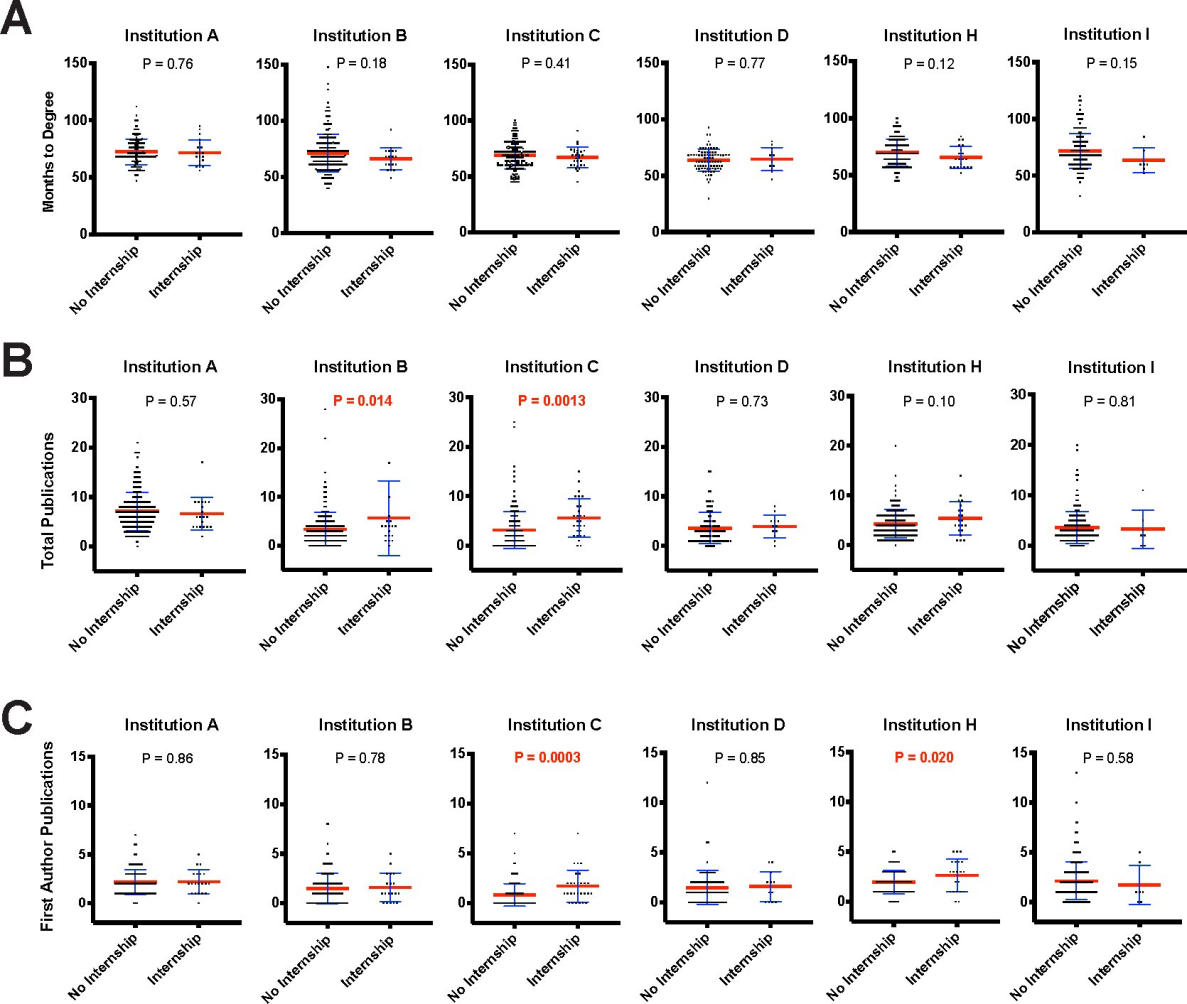
Internship or externship participation is not associated with an increase in time to degree or a decrease in total or first-author publications. **(A)** Months to degree vs. internship or externship participation. **(B)** Total publications vs. internship or externship participation. **(C)** First-author (including co-first author) publications vs. internship or externship participation. Blue error bars represent standard deviation of the mean. Mean is denoted by a red line. Independent samples *t* tests (see Tables P–R in S1 Text for statistical test results) were used to compare of control vs. participant first-author publications (significant values of *p* < 0.05 noted in red). All data sets are available at https://osf.io/qy3pa/ (permanent DOI: 10.17605/OSF.IO/QY3PA; see also [33]).

We did not detect a difference in time to degree between graduate students who completed an internship and those who did not (**Fig 7A**). This is similar to results from a graduate student internship program at the University of California San Francisco and the University of California Davis as reported by Schnoes and colleagues [43]. In addition, we found no evidence of a decrease in total publication or first-author publication productivity for individuals that participated in an internship (**Fig 7B and 7C**). Internships were associated with a favorable effect for 2 institutions’ publications. Additional data on internship participation versus weighted publication metric (**Fig D in S1 Text**) showed no effect of participation.

## Discussion

With concerns about productivity and length of doctoral education balanced with the need to provide adequate professional development, data from 10 US academic institutions were analyzed to determine if participation in career and professional development activities altered these outcomes. Here, we discuss the impact of professional development on traditional metrics of academic success.

The data show that even extensive participation did not result in a significant increase in time to degree or decreased numbers of publications for doctoral graduate students in the life sciences. Overall, this is true for both low-dose and high-dose participants generally; we found some significant changes in specific variables at some institutions as described in the results and reported in the figures.

Time to degree was chosen as a proxy for efficiency of completion because it was a measure collected at all institutions and facilitated comparisons. Publications were chosen as a proxy for productivity because they are an objective measure and because publications are widely viewed as an important indicator of graduate performance in life science higher education [44,45]. The number of publications per graduate student in this study was in alignment with prior published work, where the average publication per graduate is 2.9 publications with a range of 0 to a maximum of 16 publications [46]. Using 3 different methods of quantifying publication output (total publications, first-author publications, and the new PubMetric), we found no overall difference in publication output between participants and controls at these NIH BEST institutions.

Thus, across institutions nationwide, participating in career and professional development activities, including internships, did not negatively impact time to degree or manuscript publication. In fact, 2 institutions even showed that participants with the highest dose (internships) had the most first-author publications. Although this observation could be partly explained by the fact that this program incorporated productivity into the selection process for internships, the same institutions’ requirements for first-author publications to graduate makes this explanation unlikely. Furthermore, other internship program institutions that recommended or required a first-author publication in order for a graduate intern to be selected also typically required one or more publications to graduate, reducing the likelihood that this explanation would fully account for the potentially beneficial effect.

### Limitations

One limitation to our cross-institutional comparison is that each BEST program independently defined what it meant to be a “participant” in their program; similarly, definitions of control, low-, or high-dose participation varied by program. Three institutions defined their dosage based on the number of hours of professional development; 5 institutions defined their dosage using the number of events attended; and 2 institutions grouped their participants by the number of credits or points assigned for attendance.

Although all 17 BEST institutions were invited to participate in this study, only 10 chose to participate and had the data needed for this analysis, and, thus, the results of this study may not represent the complete impact of the program. In addition, a significant limitation of this type of analysis is the self-selection bias that exists because individual doctoral students volunteered to participate and were not randomly assigned to control and participant groups. IRB constraints limited some data sharing on the individual characteristics of the doctoral students (e.g., demographics, degree program, and pre-BEST program academic achievements); in addition, some data were not consistently available across all institutions (e.g., pre-BEST program academic achievements) so we were not able to fully assess the factors that may contribute to students selecting themselves into the program. The effect of self-selection bias could be more pronounced in highly selective application-based cohort models and competitive internship programs (programs are described in **Table E in S1 Text**). Highly motivated students may have been more likely to apply and thus more apt to succeed, but this seems unlikely to account for bias across institutions. While selective cohort models may be at a higher risk for this type of selection bias, the à la carte models were utilized so widely by trainees as to make this explanation implausible. Among the 10 institutions in this study, all offered à la carte program components to all trainees, and 4 incorporated selective components (e.g., internships). It is also possible that these selected individuals were organized multitaskers before participating and became better informed and motivated as a result of participation in BEST events.

Several of the participating institutions include PhD programs that have graduation publication requirements. In effect, this creates a floor for trainee productivity within the program. Students who are not on track to meet these requirements may be influenced by their mentor or by their own desires to graduate in a timely fashion. Such regulation of the balance between professional development and research activities could be another source of selection bias, which is not quantifiable in the current data set.

Just as the program offerings of each institution were unique, so too were the trainee populations that were eligible for programming (**Tables A–in S1 Text**). As mentioned above, most BEST institutions used an à la carte model so that trainees could choose from among professional development offerings. Others used a combination of cohort and à la carte, and some gradually opened program activities to more participants due to demand. For this reason, a classic “control” population (i.e., zero participation in professional development activities) is difficult to define when evaluating the impact of BEST programs. In addition, even the “control” population may have participated in other professional development events sponsored by other campus offices or student groups, scientific societies, companies, or other external organizations.

### Culture change

Notably, the US government clarified that researchers in doctoral and postdoctoral training who are supported by any federal funds are expected to not only conduct their research, but are also allowed to devote time to career and professional development [47]. These guidelines helped faculty to better accept the notion of doctoral students’ participation in activities outside of dissertation research.

Studies published by BEST institutions have further reinforced this change in faculty attitudes [26]. These studies showed that faculty’s initial hesitation is evolving to an understanding that next-generation scientists will not only need to be excellent researchers, but also need to be equipped with professional skills that are more effectively learned outside the laboratory. This viewpoint is supported by a snapshot of current faculty perceptions, which was obtained using subgroup surveys launched by institutions receiving NIH BEST funding. Responses showed that faculty believe that BEST career development programming is beneficial to trainees in a number of different ways: no delayed time to degree, enhanced happiness, positive effects in the lab, and more confidence in directing trainees’ own career development [26].

We predict that as additional evidence-based support for professional development comes to light, more faculty members will feel confident in encouraging their students to participate in such programming. As an example, consider the policy change of instituting rotations into the first year curriculum of PhD programs. Historically, biomedical sciences faculty expressed concerns about time spent in first year laboratory rotations when this change was first instituted, yet doctoral time to degree tracking at Cornell University revealed no statistically significant lengthening across comparison groups before and after rotations were mandated in 2003 for 3 graduate fields (**Table T in S1 Text**). Now, rotations are a widely accepted best practice within the biomedical sciences. We hope that similarly, our data will encourage widespread adoption of professional development training as an accepted foundation of PhD training.

## Conclusions and future directions

Using quantitative data collected from 10 institutions, our current study shows that participation in career exploration and professional development programming did not adversely affect time to degree or numbers of manuscripts published, and, in select cases, even correlated with more productive outcomes. We hope that the data presented herein will assuage concerns of faculty and trainees alike and will lead institutions to incorporate more experiential learning activities into PhD training programs (such as programs described in references [3,48–50]).

Academic institutions are increasingly recognizing the breadth of careers pursued by doctoral students and the need for interventions and resources to support their future success [51]. Many institutions have rapidly incorporated career and professional development training within doctoral programming [52,53]. Transformational training programs are well positioned to flourish because of this new training environment, heightened faculty awareness, institutional commitment, and support from funding agencies. Such examples are found at the Burroughs Wellcome Fund with its Career Guidance for Trainees Grant, the NIH NIGMS with its Innovative Programs to Enhance Research Training and Career Development Supplement, and the National Science Foundation with its Research Training Programs [12–14,51,54].

Although this study focused on doctoral students from biomedical fields, we anticipate that the major conclusions of this study are likely applicable to graduate students and postdoctoral researchers in other science, technology, engineering, and mathematics (STEM) fields, as well as to other fields including those in the humanities, arts, and social sciences. Nonetheless, further studies are needed to extend these conclusions across disciplines. Likewise, comparative studies between innovative biomedical PhD programs and other graduate and professional programs that have historically included embedded professional development and internship components (e.g., economics, computer science, engineering, law, to name a few) would be illuminating. Future lines of research also include measuring the long-term beneficial effect of career exploration and professional development during graduate training on such things as time in postdoctoral training; trainee mental health; time to first nontrainee job placement; fit between career exploration, skill development in training, and first career placement; career satisfaction; and salary profiles.

## Supporting information

S1 Text Figures and tables with additional information about the participating institutions and their programs, study participants, and statistical details of data presented in the main manuscript. **Fig A:** Visualization of common departments included in sample: Word cloud generator of participating departments. **Fig B:** Time to defense vs. professional development participation. **Fig C:** Weighted publication metric vs. professional development participation. **Fig D:** New publication metric vs. internship participation. **Table A:** Institutional profiles. **Table B:** BEST program activities and participating departments. **Table C:** Graduate programs/departments represented in each institution’s data set. **Table D:** Definition of dosage. **Table E:** NIH BEST programming and awardee program descriptions. **Table F:** Time to degree vs. binary BEST participation—Statistical test results. **Table G:** Time to defense vs. binary BEST participation—Statistical test results. **Table H:** Time to degree mega-analysis—Statistical test results. **Table I:** Total publications vs. professional development participation—Statistical test results. **Table J:** Total publications mega-analysis—Statistical test results. **Table K:** First-author publications vs. professional development participation—Statistical test results. **Table L:** First-author publications mega-analysis—Statistical test results. **Text A:** Publication reporting process and publication metric development. **Table M:** PubMed crawler script—Data integrity measures by institution. **Table N:** Publication metric vs. professional development participation—Statistical test results. **Table O:** Internship programs and definitions. **Table P:** Internships vs. time to degree—Statistical test results. **Table Q:** Internships vs. total publications—Statistical test results. **Table R:** Internships vs. first-author publications—Statistical test results. **Table S:** Internships vs. publication metric—Statistical test results. **Table T:** Time to degree (in years) vs. rotations at Cornell University. BEST, Broadening Experiences in Scientific Training; National Institutes of Health.
(DOCX)

## Abbreviations

ANOVA: analysis of variance
BEST: Broadening Experiences in Scientific Training
IRB: institutional review board
NIGMS: National Institute of General Medical Sciences
NIH: National Institutes of Health
NSF: National Science Foundation
STEM: science, technology, engineering, and mathematics
UNC: University of North Carolina at Chapel Hill
