# Supplementary material for: A cross-institutional analysis of the effects of broadening trainee professional development on research productivity
Source: PLoS Biol. 2021 Jul 15;19(7):e3000956. doi: 10.1371/journal.pbio.3000956 (PMC8282014; doi:10.1371/journal.pbio.3000956)
Supplement: S1 Text — Fig A: Visualization of common departments included in sample: Word cloud generator of participating departments. Fig B: Time to defense vs. professional development participation. Fig C: Weighted publication metric vs. professional development participation. Fig D: New publication metric vs. internship participation. Table A: Institutional profiles. Table B: BEST program activities and participating departments. Table C: Graduate programs/departments represented in each institution’s data set. Table D: Definition of dosage. Table E: NIH BEST programming and awardee program descriptions. Table F: Time to degree vs. binary BEST participation—Statistical test results. Table G: Time to defense vs. binary BEST participation—Statistical test results. Table H: Time to degree mega-analysis—Statistical test results. Table I: Total publications vs. professional development participation—Statistical test results. Table J: Total publications mega-analysis—Statistical test results. Table K: First-author publications vs. professional development participation—Statistical test results. Table L: First-author publications mega-analysis—Statistical test results. Text A: Publication reporting process and publication metric development. Table M: PubMed crawler script—Data integrity measures by institution. Table N: Publication metric vs. professional development participation—Statistical test results. Table O: Internship programs and definitions. Table P: Internships vs. time to degree—Statistical test results. Table Q: Internships vs. total publications—Statistical test results. Table R: Internships vs. first-author publications—Statistical test results. Table S: Internships vs. publication metric—Statistical test results. Table T: Time to degree (in years) vs. rotations at Cornell University. BEST, Broadening Experiences in Scientific Training; National Institutes of Health. (DOCX) [file pbio.3000956.s001.docx]

**S1 Figure A.** Visualization of common departments included in sample: Word cloud generator of participating departments


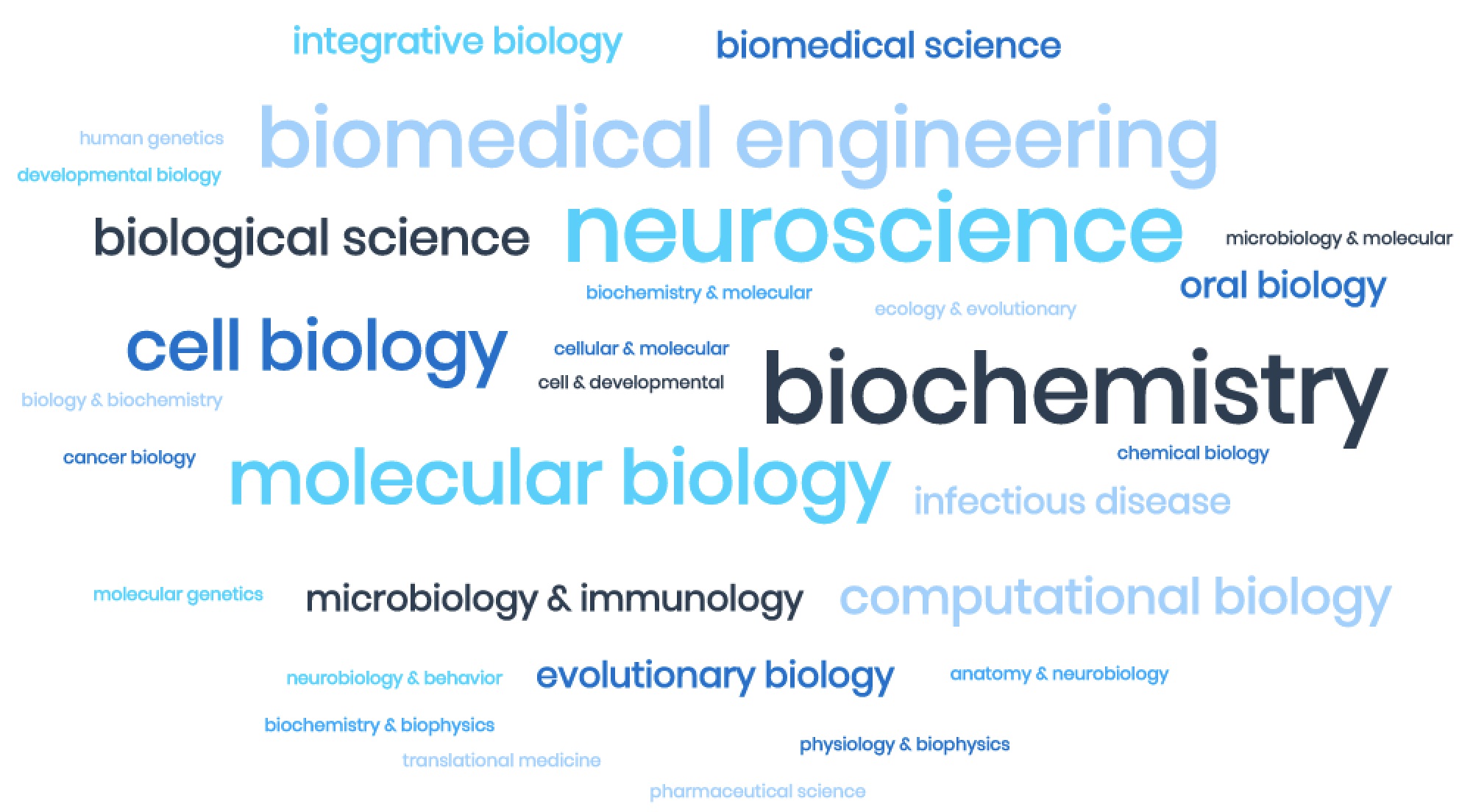


**S1 Figure B** Time to defense versus professional development participation.

Time to defense versus binary professional development participation. Blue error bars represent standard deviation of the mean. Mean is denoted by a red line. Significant p-values (<0.05) are denoted in red whereas non-significant differences are denoted in black for each independent samples t-test. B) Time to defense versus dosage of professional development participation. Blue error bars represent standard deviation of the mean. Mean is denoted by a red line. Significant p-values (<0.05) are denoted in red whereas non-significant differences are denoted in black for each institution (t-test). See S1 Table G for statistical test results. All data sets are available at <https://osf.io/qy3pa/> (permanent DOI: 10.17605/OSF.IO/QY3PA, see also reference 31).

**S1 Figure C.** Weighted publication metric versus professional development participation.

A) Weighted publication metric versus binary professional development participation. Blue error bars represent standard deviation of the mean. Mean is denoted by a red line. Significant p-values (<0.05) are denoted in red whereas non-significant differences are denoted in black for each independent samples t-test. See S1 Table N for statistical test results. B) Weighted publication metric versus dosage of professional development participation. Blue error bars represent standard deviation of the mean. Mean is denoted by a red line. Non-significant differences are denoted in black for each ANOVA (F-test). All data sets are available at <https://osf.io/qy3pa/> (permanent DOI: 10.17605/OSF.IO/QY3PA, see also reference 31).

**S1 Figure D.** New publication metric versus internship participation.

Blue error bars represent standard deviation of the mean. Mean is denoted by a red line. Non-significant differences are denoted in black for each independent samples t-test. See S1 Table S for statistical test results. All data sets are available at <https://osf.io/qy3pa/> (permanent DOI: 10.17605/OSF.IO/QY3PA, see also reference 31).

**S1 Table A.** Institutional Profiles

|  | **1** | **2** | **3** | **4** | **5** | **6** | **7** | **8** | **9** | **10** |
| --- | --- | --- | --- | --- | --- | --- | --- | --- | --- | --- |
| **Institution type** | Private | Both | Public | Public | Private | Public | Private | Private | Public | Public |
| **Campus site** | Multi campus (city) | Single campus | Multi campus | Single campus | Single campus | Single campus | Single campus | Single campus | Multi campus | Single campus |
| **Biomedical doctoral population** | 600 | 650 (3350*) | 1100 | 500 | 400 | 500 | 150 | 500 | 475 | 400 |
| **Biomedical postdoc population** | 250 | 200 (650*) | 400 | 200 | 350 | 500 | 450 | 350 | 50 | N/A |
| **Defense Pub Requirements** | Yes** | Yes** | No | Yes** | Yes** | Yes** | Yes** | Yes** | Yes** | Yes** |
| * Trainees across all discipline. ** Program dependent. *** Grad school requirements. *Note*: all institutions (appear in alphabetical order): 1) Boston University, 2) Cornell University, 3) Rutgers University, 4) University of California, Irvine, 5) University of Chicago, 6) University of North Carolina at Chapel Hill, 7) University of Rochester, 8) Vanderbilt University, 9) Virginia Tech, and 10) Wayne State University. | | | | | | | | | | |

**S1 Table B.** BEST Program Activities and Participating Departments

|  | **1** | **2** | **3** | **4** | **5** | **6** | **7** | **8** | **9** | **10** |
| --- | --- | --- | --- | --- | --- | --- | --- | --- | --- | --- |
| **Events per year** | ~70 | ~70 | ~50 | ~30 | ~50 | >90 | ~70 | ~100 | ~30 | ~30 |
| **Participation model** | Mixed | A la  carte | Mixed | Mixed | Mixed | Mixed | Mixed | A la  carte | A la  carte | Mixed |
| **Cohort competitive** | Yes –  I+S | Yes –  S | Yes –  C | No | No | Yes –  I+S | No | Yes –  I+S | No | Yes –  I |
| **Professional**  **Mentor Assigned** | No | No | Yes | Optional | No | No | No | No | No | No |
| **Prof Dev Courses Required** | Yes - Some students | No | No | No | No | No | No | No | Yes -Some programs | No |
| **I/E Requirements** | No | No | Yes  Q | No | Yes  Q | Yes  P*/Q | Yes  P/Q | Yes  Q | No | Yes  Q |
| I/E= internship or externship. Q = Qualifying Exams required for internship/externship, P= Publications required for internship/externship, P*= first-author publication recommended. “Participation model” refers to whether the BEST programs were open to all trainees (a la carte) or whether BEST programs were open to only a selected group of trainees (cohort). I+S= competitive for internships and site visits, I, S= competitive for internships or site visits only; C competitive for cohort; N/A = not applicable. Where offered, internships were always optional (opt-in) activities, as were externships, shadowing, and site visits**,** whereas professional development course were required by some. *Note*: all institutions (appear in alphabetical order): 1) Boston University, 2) Cornell University, 3) Rutgers University, 4) University of California, Irvine, 5) University of Chicago, 6) University of North Carolina at Chapel Hill, 7) University of Rochester, 8) Vanderbilt University, 9) Virginia Tech, and 10) Wayne State University. | | | | | | | | | | |

| **S1 Table C.** Graduate programs/departments represented in each institution’s dataset (listed alphabetically by institution) | | |
| --- | --- | --- |
| **Institution** | **Departments, Programs, & Fields** | |
| **Cornell University:**  *Graduate Fields (n=13)* | - Biochemistry, Molecular & Cell Biology - Biological & Environmental Engineering - Biomedical Engineering - Biophysics - Biomedical & Biological Sciences - Computational Biology - Ecology and Evolutionary Biology | - Genetics and Development - Immunology and Infectious Disease - Microbiology - Molecular and Integrative Physiology - Neurobiology and Behavior - Nutrition |
| **Boston University:**  *Programs (n=20)* | - Anatomy & Neurobiology - Behavioral Neuroscience - Biochemistry - Bioinformatics - Biology - Biomedical Engineering - Biophysics - Brain, Behavior, and Cognition - Chemistry - Genetics & Genomics | - Graduate Program for Neuroscience - Microbiology - Molecular, Cell Biology & Biochemistry - Molecular & Translational Medicine - Nutrition & Metabolism - Oral Biology - Pathology & Laboratory Medicine - Pharmacology & Experimental Therapeutics - Physiology - Program in Biomedical Sciences |
| **University of North Carolina, Chapel Hill:**  *Departments, Programs, & Curricula (n=16)* | - Bioinformatics and Computational Biology - Biochemistry and Biophysics - Biology - Biomedical Engineering - Cell Biology and Physiology - Chemistry - Genetics and Molecular Biology - Microbiology and Immunology | - Neuroscience - Nutrition - Oral Biology - Pathobiology and Translational Medicine - Pharmacology - Chemical Biology & Medicinal Chemistry - Molecular Pharmaceutics - Toxicology |
| **University of California, Irvine:**  *Departments (n=16)* | - Anatomy and Neurobiology - Biological Chemistry - Biomedical Engineering - Chemical Engineering - Chemistry - Developmental & Cell Biology - Ecology & Evolutionary Biology - Epidemiology | - Experimental Pathology - Psychiatry & Human Behavior - Microbiology and Molecular Genetics - Molecular Biology & Biochemistry - Neurobiology & Behavior - Pharmacological Sci - Physiology and Biophysics - Public Health |
| **Virginia Polytechnic Institute and State University:**  *Departments and Programs (n=9)* | - Animal and Poultry Sciences - Biochemistry - Biological Sciences - Biomedical Sciences & Pathobiology - Human Development - Human Nutrition, Foods, and Exercise - Psychology - School of Biomedical Engineering and Sciences | - Translational Biology, Medicine, and Health: - Cancer - Development, Aging, and Repair - Health Implementation Science - Immunity and Infectious Disease - Metabolic and Cardiovascular Science - Neuroscience |
| **Rutgers University:**  *Departments (n=25)* | - Cell Biology, Neuroscience and Physiology - Biomedical Engineering - Infection, Immunity and Immunology - Molecular Biology, Genetics and Cancer - Oral Biology - Biochemistry - Cell & Developmental Biology - Exposure Science - Microbiology & Molecular Genetics - Neuroscience - Pharmacology, Cellular & Molecular - Physiology & Integrative Biology | - Toxicology - Chemical & Biochemical Engineering - Chemistry & Chemical Biology - Computational Biology & Molecular Biophysics - Endocrinology Sciences - Medicinal Chemistry - Pharmaceutical Sciences - Microbial biology - Nutritional Sciences - Behavioral & Neural Sciences - Biology - Environmental Sciences - Computational & Integrative Biology |
| **University of Rochester:**  *Departments (n=16)* | - Translational - Biomedical Sciences - Biomedical Engineering - Microbiology and Immunology - Neurosciences and Neurobiology and Anatomy - Biochemistry & Biophysics - Center for Musculoskeletal Research - Brain and Cognitive Sciences | - Cardiovascular Research Institute - Ophthalmology - Environmental Medicine (Toxicology) - Biomedical Genetics - Pediatrics Infectious Diseases - Pharmacology and Physiology - Biostatistics and Computational Biology - Pathology |
| **Vanderbilt:**  *Programs & Departments (n=11)* | - Biochemistry - Biological Sciences - Cancer Biology - Cell & Developmental Biology - Cellular & Molecular Pathology - Chemical & Physical Biology | - Human Genetics - Microbiology & Immunology - Molecular Physiology & Biophysics - Neuroscience - Pharmacology |
| **Wayne State:**  *Programs & Departments (n=12)* | - Biochemistry and Molecular Biology - Biological Sciences - Biomedical Engineering - Chemistry - Immunology and Microbiology - Molecular Biology and Genetics | - Nutrition and Food Science - Pathology - Pharmaceutical Sciences - Pharmacology - Physiology - Translational Neuroscience |
| **University of Chicago:**  *Graduate programs*  *(n=17)* | - Biochemistry & Molecular Biology - Cancer Biology - Cell and Molecular Biology - Computational Neuroscience - Development, Regeneration & Stem Cell Biology - Ecology and Evolution - Evolutionary Biology - Genetics, Genomics and Systems Biology | - Public Health Sciences - Human Genetics - Immunology - Integrative Biology - Medical Physics - Microbiology - Molecular Metabolism and Nutrition - Molecular Pathogenesis and Molecular Medicine - Neurobiology |

**S1 Table D. Definition of dosage**

| **Institution** | **Control** | **Low** | **High** |
| --- | --- | --- | --- |
| **1** | 0 hours | 1-4 hours | 5+ hours |
| **2** | 0 events | 1-9 events | 10+ events |
| **3** | 0 hours | 1-39 hours | 40+ hours |
| **4** | 0/1 credits | 2-11 credits | 12+ credits |
| **5** | 0 hours | 1-11 hours | 12+ hours |
| **6** | 0 events | 1-3 events | 4+ events |
| **7** | 0 points | 5-119 points | 120+ points |
| **8** | 0 hours | 1-18 hours | 18+ hours |
| **9** | 0 events | 1-10 events | 11+ events |
| **10** | 0 events | 1-3 events | 4+ events |

Participation was recorded at each institution as hours, events, or points. All bivariate analyses compare control with any dosage (low plus high dosages combined). All dose-response analyses use the grouped definitions for control, low, and high as noted.

**S1 Table E. NIH BEST Programming & Awardee Program Descriptions**

| **Organization/ Institution** | **Description** |
| --- | --- |
| ***NIH BEST Program:*** | **Development of Programming Definitions:** Despite common data collection forms, collection procedures, and reporting methods, some inconsistencies between programs’ definitions remained, thus resulting in the convening of the BEST Consortium Data Summit, with the shared goal of establishing definitions of terms that were used in our common NIH BEST/Windrose reporting requirements. For example, there was initially confusion over what constituted a ‘workshop’ versus a ‘seminar’; consensus established at the Data Summit clarified a workshop as having a customized product or take-home material (*e.g*., personal answers to interview questions, results of a job simulation exercise, goal list, draft individual development plans; IDPs), whereas a ‘seminar’ was defined as passive listening to content delivery. Shared definitions of key terms enabled common data comparison/collection by the NIH (Lenzi et al, 2020) and have enabled collaboration between BEST institutions to compare and publish significant program outcomes in reports like this one. |
| ***Boston University BEST Program:*** | BU’s BEST is based on a classic feedback loop. Job market analysis informs program development which in turn equips trainees with the skills required to successfully join the workforce. Using the Labor Insight software tool developed by Burning Glass Technologies, jobs, job trends, job locations, and so-called “hard” and “soft” skills required for various career pathways are revealed. With this information in hand, BU's BEST offers activities to equip trainees with skills needed in six broad biomedical career tracks. The programming is designed to enable the trainees to reflect on their career interests, to explore various career paths and to enhance their skills to prepare for a productive career. Trainees are encouraged to work on an IDP to gain insight into future career possibilities while participating in coursework, workshops and panel discussions with local professionals to gain additional knowledge about options. Once trainees’ interests are refined to a particular career track, they can participate in offerings to hone the skills identified by Labor Insight. Examples of workshops include those dedicated to grant writing, data analysis, entrepreneurship, creating a successful resume, LinkedIn profile or cover letter. Site visits and internships are also offered for more experiential learning. Finally, one-on-one career coaching is available and our trainees are encouraged to utilize our alumni mentor network for informational interviews. Taken together, these tools help trainees prepare to pursue their chosen path. |
| ***Cornell University Careers Beyond Academia/BEST:*** | Cornell University’s Careers Beyond Academia/BEST provides flexible, experiential, empowering personalized opportunities for doctoral students and postdocs in all disciplines to make informed choices about their careers. Exposure to career options for PhDs comes via seminars in collaboration with department series organizers, workshops, signature “Careers in…” panels, symposia, employer site visits and courses to provide hands-on experience, all with an underlying mentoring component. Partnerships with several trainee-run associations formed and supported include the Cornell Graduate Consulting Club (CGCC), Advancing Science and Policy (ASAP), the Technology & Entrepreneurship Club (TEC), Engineering Graduate Student Association (EGSA), Chemical Biology Interface (CBI training grant) and additional student-run organizations to address programming gaps based on iterative feedback. All together we provide group and individual coaching/advising sessions, training, interactions with practitioners, case competitions, practice describing their expertise in the language of their future employer (or funder) orally and online, and practical advice on researching and obtaining a job using the skills learned. Students and postdocs are awarded funds by application to attend conferences, join professional societies beyond their academic discipline, and create their own activities which often involve alumni. Embedded in the Graduate School, we partner with the Office of Postdoctoral Studies, Entrepreneurship@Cornell, the Society for Humanities, Career Services, CU-CIRTL, the Center for Teaching Innovation and other on-campus groups to cover additional focus areas that we co-develop and co-advertise. Careers Beyond Academia/BEST enhances training opportunities for graduate students and postdoctoral scholars in all fields through an individualized, flexible program that empowers trainees to acquire the knowledge and hone skills to become more credible for an ideal career outcome. KEYS to SUCCESSES. Flexibility: a program that is ready when students and postdocs are, offering opportunities at the dose they are ready to receive, increases both faculty buy-in and trainee empowerment. Personalization: as no two careers are identical, neither will their training needs be so; we also encourage and support student/postdoc-initiated ideas. Experiential opportunities: to be able to say ‘I’ve done that’ and write it on their resume.  Gaining professorial buy-in: it is dependent on how we have marketed the program the skills learned will foster success in any field, including academia. We are not pushing students to careers beyond academia but rather are enabling an informed choice for future success. We offer a resource to alleviate faculty pain points (e.g. if they have no experience in industry, or feel they can’t connect trainees with mentors or opportunities in science policy or intellectual property law) and to showcase their successful alumni. |
| ***Rutgers iJOBS Program:*** | iJOBS (interdisciplinary Job Opportunities for Biomedical Scientists) consists of four phases and is designed to be very flexible. Phase 1 is open to all trainees and consists of weekly a la carte events to inform trainees of the various career options. Activities include hands-on job simulations, half day site visits to companies, skill primers, career panels, workshops, career fairs, and a 4-day intensive workshop SciPhD: Leadership and Business Skills for Scientists. Trainees who are interested in doing a deeper dive into a particular career track can apply to the Phase 2 cohort once they have completed at least 12 hours of Phase 1 events. About 20 trainees per year are admitted to Phase 2 and are allowed to take part in intensive training and coursework for their chosen career track. Specifically, industrial partners host unpaid shadowing/externships to allow for real world exposure (total of 72 hours over the course of a semester for observational purposes only). Trainees also register for a course to increase their knowledge base (e.g. business or law school class), and complete Individual Development Plans (IDPs) with guidance from a professional mentor. During Phase 3, trainees prepare for job search and placement via professional coaching on their resume, LinkedIn profile, and interview skills. Upon successful career placement, iJOBS program alumni are encouraged to share their wisdom with new iJOBS trainees in Phase 4 as they serve as mentors, event hosts, and shadow partners. |
| ***University of Chicago myCHOICE Program:*** | The myCHOICE program benefits from a broad-based steering committee that includes representatives from the UChicago Biological Sciences Division (BSD) postdoctoral association, graduate student dean’s council, and diversity committee, and from the UChicago biotech association, Center for teaching, Graduate Student Affairs office and Provost’s office. The myCHOICE program is open to all graduate students and postdoctoral scientists pursuing biological sciences training at UChicago. Programming emphasizes both career exposure and professional development. Career exposure covers ten general areas based on the myIDP (Individual Development Plan from Science Careers) categories. Exposure areas include such broad topics as Industry, Tech Commercialization, Entrepreneurship, Medicine/Healthcare, Business of Science, Law, Communication, Teaching, and Administration, and also provides exposure to Academic Research. myCHOICE is characterized by a three-phase training plan based around Exposure, Education and Experience (E1-E3) to the ten career areas of the above topics. The E1: Exposure seminar series “What can I do with my PhD” is open to all participants including those from other institutions. E2 and E3 level programming requires a myCHOICE application, including completion of the myIDP. UChicagoOverview: Among the unique features of the myCHOICE program also are the inclusion of guided mentorship from alumni and friends of the institution. and “outstanding leveraging of internal and external resources and the experimental nature of the plan with hypothesis testing” (quoted directly from the Resume and Summary of Discussion from peer review). Internal resources include the Booth School of Business, the Harris School of Public Policy, UChicagoTech, and the Chicago Innovation Exchange (the location of some myCHOICE events). External resources include MATTER (a Chicago based healthcare innovation center), the Alan Alda Center for Communicating Science and several local industry partners. myCHOICE benefits from a broad-based steering committee that includes representatives from the UChicago Biological Sciences Division (BSD) postdoctoral association, graduate student dean’s council, and diversity committee, and from the UChicago biotech association, Center for teaching, Graduate Student Affairs office and Provost’s office. The myCHOICE innovative evaluation plan is designed to test two hypotheses. More extensive participation in myCHOICE predicts greater PhD and postdoctoral scientist career choice empowerment and satisfaction with chosen career. More extensive participation in myCHOICE predicts improved concordance between myIDP Career Fit assessment at training exit and actual career selection. A survey of UChicago BSD graduate students after just one quarter (3 months) of myCHOICE programming has indicated that 90% of students are aware of the program and 70% have already attended at least one event. |
| ***UC Irvine GPS-BIOMED Program:*** | UCI’s Graduate Professional Success in the Biomedical Sciences (GPS-BIOMED) aims to better prepare graduate students and postdoctoral scholars for a variety of careers within the biomedical research workforce, and empower trainees to become not only skilled scientists, but also polished professionals. GPS-BIOMED is a campus-wide program spanning four schools and 20+ departments and PhD programs. Our program has a 4 pillar model: **Explore:** Increase awareness and interest in diverse science-related careers via Career nights & Life Beyond PhD seminar series. **Train**: Improve professional skills required for success in diverse fields. Science Communication Skills and Extension courses help trainees broaden the scope of career training. **Experience**: Provide hands-on experience through industry site visits, internships and externships. We offer on-campus internships as well as off-campus opportunities within local companies including, Allergan, Edwards Lifesciences, Medtronic and Evoke Neurosciences. **Transition**: Build networks that allow students and postdocs to prepare for and transition to science-related careers. Mentorship and networking are key elements for successful career transitions. With help from external advisors we host networking mixers where visitors from different job sectors discuss opportunities and daily job functions. We also have a one-on-one mentoring program.  Our program has also added value to research efforts at UCI e.g. improved scores on training grants and fellowships and new partnerships with industry. The program has helped with recruitment of talent pool. 30% of the incoming students mentioned that the presence of GPS-BIOMED program helped them make a positive decision to join UCI. Additionally, based on the alumni survey, a large percentage of them owe their career preparedness and success to GPS-BIOMED program, increasing alumni engagement. |
| **University of North Carolina at Chapel Hill TIBBS Program:** | The Training Initiatives in Biological and Biomedical Sciences (TIBBS) programming supplements our trainees scientific training with the non-bench skills needed to be successful in a wide range of careers through: regular professional development programming, student-led career cohorts, workshop series, site visits, and an immersive internship program. Professional development workshops (e.g., fellowship writing, career planning) supplements the career exposure available through the career cohorts an immersive experiences. TIBBS plans events and workshops targeted to doctoral and postdoctoral scientists at specific career stages, including TIBBS sponsors the Annual Career Blitz, which brings two-dozen scientists from a wide variety of research and research-related careers in and out of academia, to campus for an afternoon of instruction and networking (typically attended by nearly 200 scientists annually). Career cohorts focus on a wide variety of career pathways, including: business and consulting; science policy and outreach; writing and communication; teaching intensive careers; and academic and research-intensive careers. TIBBS provides structure and support for scientist-led career cohorts that meet monthly to network with invited professionals, share career resources, and report back on informational interviews. Doctoral and postdoctoral scientists gain leadership experience through their groups and groups frequently collaborate to bring in external scientists whose job duties span interest areas. Workshop series take place two to three times per year, alternating topics of interest, mirroring each of the cohort’s popular interest areas (e.g., policy series communication series, teaching series, and research-intensive careers series), as well nationally known consultants and speakers anchor the workshop series that are supplemented by expert local knowledge. as including topics spanning career areas (e.g., leadership series, and industry skills series). UNC’s proximity to the Research Triangle Park situates us to take advantage of multiple immersive learning opportunities, including site visits, and internships. Doctoral and postdoctoral scientists go on monthly field trips to local companies, organizations, and non-profits representing nearly all available career options, co-organized with local institutional partners (e.g., ELITE Consortium). TIBBS represents UNC’s long-standing and continuing commitment to professional and career development for our 1000 biological and biomedical graduate student and postdoctoral scientists. |
| ***University of Rochester URBEST Program:*** | UR’s Broadening Experiences in Scientific Training (URBEST) program funds instruction in leadership and professionalism, creates new opportunities for experiential learning through internships and shadowing, and provides training pathways in (1) industry, manufacturing and entrepreneurship; (2) regulatory affairs, compliance and review; and (3) science and technology policy. First URBEST program activities include an Individual Development Plan (IDP) Workshop, a Leadership Advantage Program, and a URBEST Retreat and Career Workshop. Internship opportunities are available to select URBEST participants who show research productivity and career development initiative. Novel components of the URBEST program include: Incorporating Self-Determination Theory (SDT) into IDPs and program evaluations to ensure continuous improvement and effectiveness of URBEST activities. Faculty, alumni and peer mentoring networks have been established using a URBEST LinkedIn Group and SDT-based Mentoring Workshops to foster improved mentoring practices and better support scientist autonomy and diverse career outcomes. Other activities include a flipped-classroom Career Stories Q&A Seminar series, and new courses tailored for URBEST participants, such as an Intellectual Property and Commercializing Technology Seminar Series and a Leadership and Management for Scientists Course. |
| **Vanderbilt ASPIRE Program:** | The ASPIRE Program is designed to empower Vanderbilt’s biomedical sciences PhD students and postdoctoral scholars to make well-informed career decisions with confidence. ASPIRE provides PhD students and postdocs (collectively, called “trainees”) with programs for professional development, career exploration, and career enhancement. Except for a few professional development sessions that are required for all first year PhD students, all other activities are optional and trainees choose from among the offerings according to their training stage and career interests. Professional development opportunities include a twice-monthly ASPIRE Postdoctoral Café series for postdocs, an annual ½ day ASPIRE to Connect workshop focused on the importance of building professional relationships, a series of career planning sessions for first year students or more advanced grad students and postdocs, and several non-credit bearing short courses in communication-related topics. Career exploration activities include a collection of nearly 100 Beyond the Lab interviews with Vanderbilt PhD and postdoctoral alumni discussing their careers (<https://medschool.vanderbilt.edu/career-development/beyond-the-lab-see-listen/>), a monthly PhD Career Stories seminar series, and an Annual Career Symposium. Career enhancement activities are intended for post-qualifying PhD students and postdoctoral fellows, and include opportunities to participate in didactic and experiential modules, the opportunity to gain hands-on-experience through ASPIRE internships, and the opportunity to gain deeper insight into specific industries through ASPIRE on the Road group field trips to visit cities with high concentrations of biotech or policy-related employers. For a description of the full range of ASPIRE program features, see the ASPIRE Annual Reports at <https://medschool.vanderbilt.edu/career-development/annual-report/>. |
| **Virginia Tech BEST Program:** | Virginia Tech’s Broadening Experiences in Scientific Training (BEST) Program activities are open to participation by pre- and postdoctoral scientists at any stage of their training, with some activities required by one or more graduate programs. Core offerings include: I. A 2-credit professional development course focused on self-assessment, skill-building, and career pathways for biomedical PhDs. Example skills topics include grantsmanship and CV writing, improvisation for science communication, and budgeting. II. Individual Development Planning workshops, III. Job simulation workshops delivered by outside professionals from a variety of careers, which involve hands-on activities and case studies. IV. A commercialization/shark tank module and pitch competition run jointly with biomedical engineering and business faculty. VT-BEST also delivers one-off activities such as networking training preceding professional/scientific events (with VT’s Career Center), and workshops such as visualizing data or social media for scientists (with VT’s Center for Communicating Science). Virginia Tech’s recent partnership with the Roanoke RAMP accelerator provides training and shadowing opportunities in commercialization and start-ups. VT-BEST also facilitates a small number of internship opportunities, through industry partnerships and travel awards. Lastly, VT-BEST staff work closely with the Roanoke Graduate Student Association, Virginia Tech Carilion Student Outreach Program, and individual trainees, on the implementation of trainee-driven professional development activities. |
| **Wayne State BEST Program:** | Wayne State BEST assists doctoral students in exploring and pursuing a variety of career options. One of the highlights of Wayne State BEST is that doctoral students outside the biomedical disciplines also participate, adding richness to the training experiences. Wayne State BEST has three successive phases: Phase I – Exploratory Seminars; Phase II – Didactic Workshops; Phase III – Career Exploration/Internships. Phase I acquaints students with multiple career options via 90-minute seminars, each exploring one of five career tracks with industry partners, faculty, and alumni whose work intersects with the biosciences and the following areas: undergraduate teaching, law, communication, business/industry, and government. Phase II comprises a series of daylong workshops on the career options identified in Phase I. These workshops serve as a bridge between the Phase I exploratory seminars and the Phase III career exploration/internship experiences. A team comprising community and industry leaders (including alumni) work with faculty facilitators to design a curriculum focusing on necessary skills sets for each of the career tracks. Attendees gain additional knowledge through one-on-one exchanges with professionals in these domains. Students learn how their scientific training, problem-solving abilities, and analytical aptitude can be mobilized to successfully address the needs of their desired career. Phase III offers students experiential learning about these career paths through career explorations/internships with private industry, state agencies, nonprofit organizations, or primarily undergraduate institutions. Wayne State offers workshops on constructing an Individualized Development Plan (IDP), which is required of all doctoral students. In addition, the Wayne State Graduate School offers professional development seminars on basic employment skills such as conducting a job search, preparing for an interview, converting a CV to a resume, building a LinkedIn page, and writing a cover letter. Wayne State faculty lead specialized workshops on abstract writing, poster presentation, professional communication in the workplace, and strategies for presenting scientific ideas to non-specialist audiences. The Graduate School established a 1-credit course for graduate students interested in preparing for a career outside of academia. This course uses exercises and assignments to build a professional portfolio necessary for employment in highly skilled positions. |

**S1 Table F.** Time to degree versus binary BEST participation (N_TOTAL_ = 1742). Statistical test results.

| **Institution** | **Mean CTRL Degree** | **Mean BEST Degree** | **R^2^** | **t-test** | **p-Value** | **N CTRL** | **N BEST** |
| --- | --- | --- | --- | --- | --- | --- | --- |
| Institution A | 74.79 | 71.44 | 0.02 | -1.92 | 0.06 | 52 | 197 |
| Institution B | 73.09 | 69.01 | 0.02 | -1.89 | 0.06 | 101 | 136 |
| Institution C | 68.69 | 67.90 | 0.02 | -0.50 | 0.62 | 121 | 102 |
| Institution D | 62.16 | 64.69 | 0.02 | 1.11 | 0.27 | 26 | 69 |
| Institution E | 51.94 | 58.36 | 0.02 | 1.45 | 0.15 | 79 | 20 |
| Institution F | 68.44 | 69.32 | <.01 | 0.33 | 0.74 | 97 | 97 |
| Institution G | 68.93 | 64.30 | 0.01 | -0.95 | 0.34 | 27 | 59 |
| **Institution H** | **73.08** | **67.71** | **0.04** | **-2.76** | **<.01** | **50** | **126** |
| **Institution I** | **74.03** | **69.49** | **0.02** | **-2.57** | **0.01** | **145** | **147** |
| Institution J | 63.00 | 65.73 | 0.05 | 0.98 | 0.33 | 24 | 67 |

**S1 Table G.** Time to defense versus binary BEST participation (N_TOTAL_ = 1336). Statistical test results.

| **Institution** | **Mean CTRL Defense** | **Mean BEST Defense** | **R^2^** | **t-test** | **p-Value** | **N CTRL** | **N BEST** |
| --- | --- | --- | --- | --- | --- | --- | --- |
| Institution A | 72.08 | 68.97 | 0.02 | -1.80 | 0.07 | 52 | 197 |
| **Institution B** | **71.62** | **65.26** | **0.04** | **-2.86** | **<.01** | **82** | **127** |
| Institution C | 66.13 | 65.82 | 0.02 | -0.20 | 0.84 | 121 | 102 |
| Institution F | 65.37 | 66.71 | <.01 | 0.51 | 0.61 | 97 | 97 |
| **Institution H** | **69.78** | **65.17** | **0.03** | **-2.43** | **0.02** | **50** | **126** |
| Institution I | 67.09 | 66.30 | <.01 | -0.52 | 0.60 | 140 | 145 |

**S1 Table H.** Time to Degree Mega-Analysis. Statistical test results.

|  |
| --- |

| *Estimates* | | | | | |
| --- | --- | --- | --- | --- | --- |
| *Label* | *Estimate* | *Standard Error* | *DF* | *t Value* | *Pr > \|t\|* |
| *Control* | 67.9377 | 2.0281 | 9 | 33.50 | <.0001 |
| *Intervention 1 (High)* | 67.0765 | 2.1149 | 9 | 31.72 | <.0001 |
| *Intervention 2 (Low)* | 65.5995 | 2.1514 | 9 | 30.49 | <.0001 |
| *Intervention 1 (High) vs. Control* | -0.8612 | 0.9802 | 9 | -0.88 | 0.4025 |
| *Intervention 2 (Low) vs. Control* | -2.3383 | 1.0529 | 9 | -2.22 | 0.0535 |
| *Intervention 2 (High) vs. Intervention 1 (Low)* | -1.4771 | 0.8908 | 9 | -1.66 | 0.1317 |
| *Control vs. Intervention (High +Low)* | -1.5997 | 0.9145 | 9 | -1.75 | 0.1142 |

| *Contrasts* | | | | |
| --- | --- | --- | --- | --- |
| *Label* | *Num DF* | *Den DF* | *F Value* | *Pr > F* |
| Total | 2 | 9 | 2.66 | 0.1234 |

**S1 Table I**. Total Publications versus Professional Development Participation. Statistical test results.

| **Institution** | **Mean CTRL Pubs** | **Mean BEST Pubs** | **R^2^** | **t-test** | **p-Value** | **N CTRL** | **N BEST** |
| --- | --- | --- | --- | --- | --- | --- | --- |
| Institution A | 7.63 | 6.90 | <.01 | -1.23 | 0.22 | 52 | 197 |
| **Institution B** | **2.94** | **4.07** | **0.02** | **2.14** | **0.03** | **101** | **136** |
| Institution C | 3.11 | 3.57 | 0.01 | 0.90 | 0.37 | 80 | 196 |
| Institution D | 3.50 | 3.62 | 0.02 | 0.17 | 0.87 | 26 | 71 |
| Institution E | 3.43 | 3.62 | 0.05 | 0.17 | 0.87 | 79 | 20 |
| **Institution F** | **7.86** | **5.90** | **0.03** | **-2.07** | **0.04** | **97** | **97** |
| Institution G | 6.12 | 5.06 | 0.02 | -1.17 | 0.25 | 25 | 52 |
| Institution H | 5.00 | 4.15 | 0.02 | -1.74 | 0.08 | 50 | 126 |
| Institution I | 3.59 | 3.54 | <.01 | -0.14 | 0.89 | 145 | 148 |

**S1 Table J.** Total Publications Mega-Analysis**.** Statistical test results.

| \| *Contrasts* \| \| \| \| \| \| --- \| --- \| --- \| --- \| --- \| \| *Label* \| *Num DF* \| *Den DF* \| *F Value* \| *Pr > F* \| \| Total \| 2 \| 8 \| 0.24 \| 0.7898 \| | | | | | | |
| --- | --- | --- | --- | --- | --- | --- | --- | --- | --- | --- | --- | --- | --- | --- | --- | --- | --- | --- | --- | --- | --- |
| *Label* | *Estimate* | *Standard Error* | *DF* | *t Value* | *Pr > \|t\|* |  |
| *Control* | 4.6724 | 0.5382 | 8 | 8.68 | <.0001 |  |
| *Intervention 1 (Low)* | 4.6691 | 0.5598 | 8 | 8.34 | <.0001 |  |
| *Intervention 2 (High)* | 4.5058 | 0.5715 | 8 | 7.88 | <.0001 |  |
| *Intervention 1 (Low) vs. Control* | -0.00338 | 0.2671 | 8 | -0.01 | 0.9902 |  |
| *Intervention 2 (High) vs. Control* | -0.1666 | 0.2888 | 8 | -0.58 | 0.5798 |  |
| *Intervention 2 (High) vs. Intervention 1 (Low)* | -0.1633 | 0.2566 | 8 | -0.64 | 0.5423 |  |
| *Control vs. Intervention (High + Low)* | -0.08501 | 0.2468 | 8 | -0.34 | 0.7394 |  |

**S1 Table K**. First-author Publications versus Professional Development Participation. Statistical test results.

| **Institution** | **Mean CTRL FA Pubs** | **Mean BEST FA Pubs** | **R^2^** | **t-test** | **p-Value** | **N CTRL** | **N BEST** |
| --- | --- | --- | --- | --- | --- | --- | --- |
| Institution A | 2.37 | 2.10 | <.01 | -1.34 | 0.18 | 52 | 197 |
| Institution B | 1.35 | 1.62 | <.01 | 1.31 | 0.19 | 101 | 136 |
| **Institution C** | **0.66** | **1.05** | **0.04** | **2.43** | **0.02** | **80** | **196** |
| Institution D | 1.65 | 1.40 | 0.02 | -0.65 | 0.51 | 26 | 71 |
| Institution E | 1.73 | 1.63 | <.01 | -0.18 | 0.86 | 79 | 20 |
| **Institution F** | **2.16** | **1.57** | **0.03** | **-2.25** | **0.03** | **97** | **97** |
| Institution G | 2.68 | 2.03 | 0.02 | -1.21 | 0.23 | 25 | 52 |
| Institution H | 2.00 | 2.03 | <.01 | 0.15 | 0.88 | 50 | 126 |
| Institution I | 2.10 | 2.11 | 0.02 | 0.03 | 0.98 | 145 | 148 |

**S1 Table L.** First Author Publications Mega-Analysis**.** Statistical test results.

| *Estimates* | | | | | |
| --- | --- | --- | --- | --- | --- |
| *Label* | *Estimate* | *Standard Error* | *DF* | *t Value* | *Pr > \|t\|* |
| *Control* | 1.7878 | 0.1707 | 8 | 10.47 | <.0001 |
| *Intervention 1 (Low)* | 1.7717 | 0.1855 | 8 | 9.55 | <.0001 |
| *Intervention 2 (High)* | 1.7534 | 0.1923 | 8 | 9.12 | <.0001 |
| *Intervention 1 (Low) vs. Control* | -0.01601 | 0.1161 | 8 | -0.14 | 0.8937 |
| *Intervention 2 (High) vs. Control* | -0.03433 | 0.1254 | 8 | -0.27 | 0.7913 |
| *Intervention 2 (High) vs. Intervention 1 (Low)* | -0.01832 | 0.1274 | 8 | -0.14 | 0.8893 |
| *Control vs. Intervention (High + Low)* | -0.02517 | 0.1027 | 8 | -0.25 | 0.8126 |

| *Contrasts* | | | | |
| --- | --- | --- | --- | --- |
| *Label* | *Num DF* | *Den DF* | *F Value* | *Pr > F* |
| Total | 2 | 8 | 0.04 | 0.9633 |

S1 Text A. Publication reporting and publication metric development

**Publication data collection procedures**

First-author and co-first-author publications were included in the first-author publication count. Publications and their metadata were primarily collected through a Python script built to query the PubMed API (reference 32), in combination with manual verification (see **S1 Table M**). By automating the PubMed search process, the script allowed for replication and validation of publication data across multiple institutions and implementation of Cross-Institutional Instructions. Manual checking was used for institutions that could not access PubMetric results for technical reasons (one of ten institutions); or existing survey data from a graduate school survey were used (one of ten institutions).

**Publication metric**

The metric is a weighted calculation of different types of publications determined by polling 375 active training faculty at UNC about the relative value they place on different publication types. Respondents (n=120) were asked to assign their values to the following types of publications:

o   First-author (and co-first-author) research paper (FA Res)

o   Middle-author research paper (MA Res)

o   First-author review (FA Rev)

o   Middle-author review (MA Rev)

Based on the responses on a scale of 1-10 (1=*Not valuable*, 10=*Extremely valuable*), an average rating was calculated for each type of publication. Once all four of the most common metrics were calculated, a weighted publication metric was created to represent the productivity of any given trainee. The metric is given by the equation: 2.07*Number of First-author Research papers + 1.37*Number of Middle-author Research papers + 1.54*Number of First-author Reviews +1*Number of Middle-author Review papers.

The new weighted pub metric developed based on the average of 120 responses was as follows:

= 2.07*(FA Res)+1.37*(MA Res)+1.54*(FA Rev)+1*(MA Rev)

This allows for the use of a single publication metric rather than having to depend on multiple measures, and may be of use especially when simplicity or overall trends are of most interest. This method of evaluating productivity is an alternative to attempting to assign credit to various flagship journals by name (which can be difficult to capture across fields), or impact factor measures (which are controversial), and provides an independent estimate of productivity based on role/contribution to each work as well as accounting for type of publication.

**Sample survey**

**SURVEY:** **Faculty survey to create publication productivity rating**

Start of Block: Productivity Metric Survey

As part of a project to examine graduate student productivity we need your input. Your response will be used to develop a new metric to represent trainee publication “productivity” as a single quantitative measure.

Please rate the relative value you give to each publication type when evaluating trainee productivity.

---

Q: How would you value the contribution of a candidate with each of the following publication types?

| 1 - Not valuable | 2 | 3 | 4 | 5 | 6 | 7 | 8 | 9 | 10- Extremely Valuable |
| --- | --- | --- | --- | --- | --- | --- | --- | --- | --- |

First-Author Peer-Reviewed Scientific Publication

Middle-Author Peer-Reviewed Scientific Publication

First-Author Review Article or Book Chapter (Peer Reviewed)

Middle-Author Review Article or Book Chapter (Peer Reviewed)

Other publication type?(specify or skip)

Other publication type? (specify or skip)

Other publication type? (specify or skip)

---

Q: Have you supervised graduate or post-doctoral trainees? (Yes/No)

---

Q: How many total graduate student and post-doctoral trainees have you supervised?

Number of trainees (total)

| 0 | 5 | 10 | 15 | 20 | 25 |
| --- | --- | --- | --- | --- | --- |

----

Thank you for helping to develop a new metric for assessing graduate student productivity. We will share the survey results with all respondents after the survey closes on 12/6/17.

**---**

**S1 Table M**. PubMed Crawler Script – Data Integrity Measures by Institution

| **Institution** | **Publication Data Collection Method** | **Data check method** | **Note** |
| --- | --- | --- | --- |
| Institution A | Algorithm | Manual checks | Checked for 0 & > 4 |
| Institution B | Algorithm | Manual checks | Checked for 0 & > 4 |
| Institution C | Algorithm | Checked all | Checked all |
| Institution D | Algorithm | Checked all | Checked all |
| Institution E | Manual Lookup | Checked all | Checked all |
| Institution F | Surveys | Self-report | Student exit survey |
| Institution G | Algorithm | Checked all | Checked all |
| Institution H | Internal Algorithm | Manual checks | Checked for 0 and ≥15  Common names verified |
| Institution I | Algorithm + Internal Algorithm | Manual checks | Checked for 0 & > 20  90 algorithm  120 internal algorithm |

**S1 Table N**. Publication Metric versus Professional Development Participation. Statistical test results.

| **Institution** | **Mean CTRL Pub Metric** | **Mean BEST Pub Metric** | **R^2^** | **t-test** | **p-Value** | **N CTRL** | **N BEST** |
| --- | --- | --- | --- | --- | --- | --- | --- |
| Institution A | 8.65 | 7.86 | <.01 | -1.14 | 0.26 | 52 | 197 |
| Institution C | 3.73 | 4.07 | <.01 | 0.59 | 0.56 | 80 | 196 |
| Institution E | 5.81 | 5.81 | 0.03 | 0.00 | 1.00 | 79 | 20 |
| **Institution F** | **9.18** | **6.78** | **0.03** | **-2.09** | **0.04** | **97** | **97** |
| Institution H | 8.05 | 6.94 | 0.01 | -1.49 | 0.14 | 50 | 126 |
| Institution I | 4.05 | 4.10 | 0.02 | 0.12 | 0.91 | 145 | 148 |

**S1 Table O.** Internship programs and definitions

| **Organization/ Institution** | **Experiential Learning Program Description** |
| --- | --- |
| ***NIH BEST Program:*** | Following the BEST Data Summit an internship is defined as working in a professional setting for the purpose of receiving *hands-on* *training*. An internship assumes the trainee is able to develop some skills during the experience and results in a deliverable. On the other hand, an externship is defined as job shadowing a professional at work for the purpose of *observing and* *experiencing* the work environment and learning about the expectations of a profession (Schnoes, et al. 2018. Ref 43 in manuscript). In both an internship and externship, significant time is spent in the professional workplace environment and therefore out of the graduate student’s own laboratory. For the purposes of this paper, internship and externship are collectively referred to as internship for consistency. |
| ***Boston University BEST Program:*** | BU’s BEST program established relationships with departments and programs within BU and with local employers and nonprofit organizations to develop internship opportunities in diverse career tracks such as business, administration, communication, policy, research and teaching. Sites submitted a description of the internship including intern responsibilities, deliverable and its evaluation, professional development objectives, benefits to the intern, and assigned mentor. Internships were offered on a rolling basis, varied both in length (from 1 month to 1 year) and in time (from 1-40 hours/week), and were paid where possible. Applicants must have passed their qualifying exams prior to the start of an internship, have a completed IDP on file, and have attended basic skill building pre-internship workshops (e.g. professionalism 101) and internship-specific workshops. Applicants submitted their resume, approval by the research advisor, sign off on satisfactory academic performance, a personality assessment and a pre-internship evaluation. Each applicant met individually with the internship director prior and after applying to ensure that the trainee’s career goals were aligned with the goals of the internship. BU’s BEST presented qualified applicants to the internship site. Interns were selected by the employers, and developed projects with deliverables set by the internship site. Both interns and site met with the internship director to evaluate learning objectives mid- and end of the internship. |
| ***Rutgers iJOBS Program:*** | Rutgers trainees who are interested in doing a deeper dive into a particular career track can apply to the Phase 2 cohort once they have completed at least 12 hours of Phase 1 events and completed their qualifying exams. About 20 trainees per year are admitted to Phase 2 and are matched by the iJOBS program directors with a professional in their area of interest for an externship/shadowing experience. The trainees spend time in the professional’s workplace sitting in on meetings and observing their activities for a total of 72 hours spread out over the course of a semester. |
| ***University of Chicago myCHOICE Program:*** | A key goal of myCHOICE Experience programming is to provide real-world, practical experience in a specific career field. Internships, defined as “hands-on opportunities of limited duration (weeks to months),” are an important component of this training experience. myCHOICE collaborates with on- and off-campus partners to develop a diverse array of internships varying from scientific writing, to investment banking, to marketing and program development. All myCHOICE internships are unpaid and part-time (10 hours per week), lasting approximately 10 weeks. Trainees interested in internships must apply and receive permission from their PI. Interns who are graduate students formally register for the internship and receive academic credit. |
| ***University of North Carolina at Chapel Hill TIBBS Program:*** | UNC’s ImPACT Internship program consisted of 160-hours, typically completed either one-month full time or multi-month part-time, in the career field of choice (industry research and development, science policy, teaching, museums/outreach, startups, etc.) Approximately 25-30 interns per year are typically selected to participate based on training stage (comps/quals complete to reach candidacy stage, often fourth or fifth years of training); research status (appropriate progress toward or completion of first-author publication for training stage); career exploration and professional development training; and selection of career path with competitive skills appropriate for field selected. This is the capstone experience available to UNC graduate students on a competitive basis are 160-hour internships that can take place during 1 month of full-time effort, or part time over 2-3 months. Interns are paid at their current stipend of salary rate. Graduate students must have passed their qualifying exams and all scientists must have written support of their faculty mentor in order to apply. |
| ***University of Rochester URBEST Program:*** | URBEST developed a flexible experiential learning program that included long-term internships (full-time, up to three month), short-term internships (hours-per-week) shadowing experiences (a couple of days total) and volunteer opportunities (< 4 hours per week). Internships took place within University of Rochester at core facilities (e.g., Office of Regulatory Support, Upstate Stem Cell Good Manufacturing Practice (GMP) Facility, Flow Cytometry Core) or within the city of Rochester (e.g., Rochester Museum and Science Center, Litron Laboratories). The majority of URBEST internships took place in different cities (e.g., Entasis Therapeutics, The US Food and Drug Administration (FDA), Pfizer Vaccines). To be allowed to do an internship, the learner must have officially enrolled in URBEST as a trainee for at least 6 months, have collected ~ 40 – 60 points through the program, have passed their qualifying exam and have the permission of their PI. The graduate student was also required to have a first author publication or multiple publications if they were not first author. The URBEST program disseminated intern opportunities as they became available, posting them first to our URBEST LinkedIn Group as a benefit of enrolling in the program. While undertaking their internship all trainees needed to be registered as a PhD graduate student. If the graduate student was on some type of training grant or fellowship, they needed to discuss your stipend and training opportunity with their program officer to get approval for their internship. It is up to the program officer as to whether or not their internship contributed to graduate student training, during the URBEST program all program officers approved graduate student internship requests. Most internships were set up by the trainee using a “cold email” technique to set up informational interviews, which often led to experiential opportunities. A few trainees found well established internships that they could also apply (e.g., Scientific American, Bayer Global Regulatory Affairs, White House Office of Science and Technology Policy). |
| ***Vanderbilt ASPIRE Program:*** | Vanderbilt University’s ASPIRE program has established relationships with local employers and several national nonprofit organizations to develop part-time internship opportunities in a range of career areas, including data science, college teaching, nonprofit management, business development, marketing, science policy and advocacy, and science outreach. ASPIRE internships are paid where possible, part-time (usually 6-8 hours per week), and generally last 10-12 weeks. Internships are offered on a rolling basis according to employer need and desired timing. All PhD student interns must have passed their qualifying exams prior to the start of an internship, and each applicant meets individually with our office staff prior to applying to ensure that the trainee’s career interests are aligned with the goals of the internship. Interns are selected by the employers, and interns are expected to contribute to one or more projects during their internship. Since the inception of the ASPIRE internship program in 2015, nearly 100 trainees have completed internships, about 65% of whom have been PhD students. |

**S1 Table P.** Internships versus time to degree. Statistical test results.

| **Institution** | **Mean CTRL Degree** | **Mean INTERNSHIP Degree** | **R^2^** | **t-test** | **p-Value** | **N CTRL** | **N INTERNSHIP** |
| --- | --- | --- | --- | --- | --- | --- | --- |
| Institution A | 72.30 | 71.50 | <.01 | -0.31 | 0.76 | 229 | 20 |
| Institution B | 71.34 | 66.16 | <.01 | -1.34 | 0.18 | 218 | 19 |
| Institution C | 68.88 | 66.93 | <.01 | -0.82 | 0.41 | 195 | 28 |
| Institution D | 63.81 | 64.73 | <.01 | 0.30 | 0.77 | 83 | 12 |
| Institution H | 69.76 | 65.48 | 0.01 | -1.57 | 0.12 | 155 | 21 |
| Institution I | 71.94 | 63.43 | <.01 | -1.46 | 0.15 | 285 | 7 |

**S1 Table Q.** Internships versus total publications. Statistical test results.

| **Institution** | **Mean CTRL Pubs** | **Mean INTERNSHIP Pubs** | **R^2^** | **t-test** | **p-Value** | **N CTRL** | **N INTERNSHIP** |
| --- | --- | --- | --- | --- | --- | --- | --- |
| Institution A | 7.10 | 6.60 | <.01 | -0.57 | 0.57 | 229 | 20 |
| **Institution B** | **3.37** | **5.68** | **0.03** | **2.48** | **0.01** | **218** | **19** |
| **Institution C** | **3.16** | **5.61** | **0.04** | **3.25** | **<.01** | **248** | **28** |
| Institution D | 3.59 | 3.92 | <.01 | 0.34 | 0.73 | 85 | 12 |
| Institution H | 4.26 | 5.38 | 0.02 | 1.65 | 0.10 | 155 | 21 |
| Institution I | 3.58 | 3.29 | <.01 | -0.24 | 0.81 | 286 | 7 |

**S1 Table R.** Internships versus first-author publications. Statistical test results.

| **Institution** | **Mean CTRL FA Pubs** | **Mean INTERNSHIP FA Pubs** | **R^2^** | **t-test** | **p-Value** | **N CTRL** | **N INTERNSHIP** |
| --- | --- | --- | --- | --- | --- | --- | --- |
| Institution A | 2.15 | 2.20 | <.01 | 0.17 | 0.86 | 229 | 20 |
| Institution B | 1.48 | 1.58 | <.01 | 0.28 | 0.78 | 218 | 19 |
| **Institution C** | **0.83** | **1.71** | **0.05** | **3.71** | **<.01** | **248** | **28** |
| Institution D | 1.48 | 1.58 | <.01 | 0.19 | 0.85 | 85 | 12 |
| **Institution H** | **1.94** | **2.62** | **0.03** | **2.34** | **0.02** | **155** | **21** |
| Institution I | 2.12 | 1.71 | <.01 | -0.56 | 0.58 | 286 | 7 |

**S1 Table S.** Internships versus publication metric. Statistical test results.

| **Institution** | **Mean CTRL Pub Metric** | **Mean INTERNSHIP Pub Metric** | **R^2^** | **t-test** | **p-Value** | **N CTRL** | **N INTERNSHIP** |
| --- | --- | --- | --- | --- | --- | --- | --- |
| Institution A | 8.10 | 7.36 | <.01 | -0.71 | 0.48 | 229 | 20 |
| **Institution C** | **3.66** | **6.35** | **0.03** | **3.08** | **<.01** | **248** | **28** |
| Institution H | 7.04 | 8.86 | 0.02 | 1.76 | 0.08 | 155 | 21 |
| Institution I | 4.10 | 3.37 | <.01 | -0.56 | 0.57 | 286 | 7 |

**S1 Table T**. Time to degree (in years) versus rotations at Cornell University

|  | **Before rotations (2003)** | | **After mandated rotations (2003-2014)** | |
| --- | --- | --- | --- | --- |
|  | **Median TTD** | ***n*** | **Median TTD** | ***n*** |
| **Biomedical and Biological Sciences** | 5.0 | (107) | 5.1 | (82) |
| **Molecular & Integrative Physiology** | 5.4 | (46) | 5.4 | (30) |
| **Pharmacology** | 6.2 | (31) | 6.0 | (23) |
| **Overall Median (total PhD graduates)** | 5.3 | (184) | 5.4 | (135) |

Data analyzed from Cornell University revealed no statistically significant lengthening of degree across comparison groups before and after rotations were mandated in 2003 for three graduate fields. Independent samples t-tests (*t* = 0.80, *p* = NS) do not show a significant difference in TTD before and after mandated rotation.
